# Design and synthesis of benzothiazole/thiophene-4*H*-chromene hybrids†

**DOI:** 10.1039/c8ra08262f

**Published:** 2018-12-13

**Authors:** Lakshmanan Pazhanivel, Vasuki Gnanasambandam

**Affiliations:** Department of Chemistry, Pondicherry University Pondicherry 605 014 India vasukig@gmail.com

## Abstract

A library of 4*H*-chromene derivatives with heterocyclic substituent's at the 3 and 4-positions was synthesized in a convenient DBU catalysed three component synthesis between salicylaldehyde, acetonitrile derivatives and thiazolidinedione to afford 2-amino-3-benzothiazole-4-heterocycle-4*H*-chromenes and 2-amino-3-thiophenoyl-4-heterocycle-4*H*-chromenes derivatives in ethanol and a mixture of ethanol and water (1 : 1) at room temperature. The significance of this protocol is the feasibility of incorporating substituents simultaneously at the 3 and 4 positions of 4*H*-chromenes in an efficient three component reaction.

## Abstract 2

A library of 4*H*-chromene derivatives with heterocyclic substituents at 3 and 4-positions was synthesized in a convenient DBU catalysed three component reaction.

## Graphical abstract

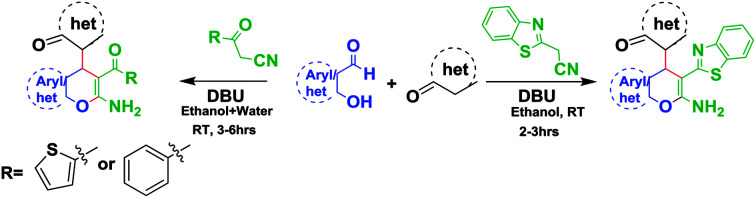

## Introduction

The design of highly functionalized small organic molecules with features suited for highly selective binding to macromolecules is crucial to accelerate the drug discovery process.^1^ Therefore; the synthesis of molecules that are enriched with therapeutic values has become the main objective for organic and medicinal chemistry research projects. In the process of developing new potent small molecules, there is an approach where two or more bioactive heterocyclic scaffolds are embedded in a single molecule to access heterocyclic hybrid molecules which are hoped to exhibit enhanced activity with different kind of action.^2^

4*H*-Chromene derivatives,^3^ as a privileged heterocyclic scaffold of medicinal importance have attracted medicinal chemists, due to their wide range of biological and pharmacological properties. In particular, 4-substituted-4*H*-chromenes have attracted wide attention for their remarkable anti-cancer activity.^4–10^

Owing to its diverse biological applications,^11^ the benzothiazole core represents an ideal source for medicinal chemists in designing new therapeutic agents which may allow access to unexplored areas of biologically relevant chemical space. A wide range of benzothiazole derivatives were found to possess anticancer activity, and there were several reports, where the benzothiazole nucleus was modified in order to improve their antitumor activities.^12–20^

The anticancer activity of these molecules may be attributed to the formation of reactive intermediates that can bind covalently to DNA.^21^ Modifications on the benzothiazole nucleus have resulted in a large number of hybrids having diverse pharmacological activities. The excellent antitumour potential of these hybrid molecules have attracted our attention to synthesize heterocyclic hybrids of benzothiazole and 4-substituted chromene template. Due to their diverse biological activities, many of the thiophene derivatives are widely used as therapeutic agents as anticancer agents against various cancer cell lines.^22–27^ Therefore, it was planned to synthesize the thiophene/benzothiazole-4*H*-chromene hybrid with a view that the designed heterocycle hybrids may possess enhanced activity.

**Fig. 1:**
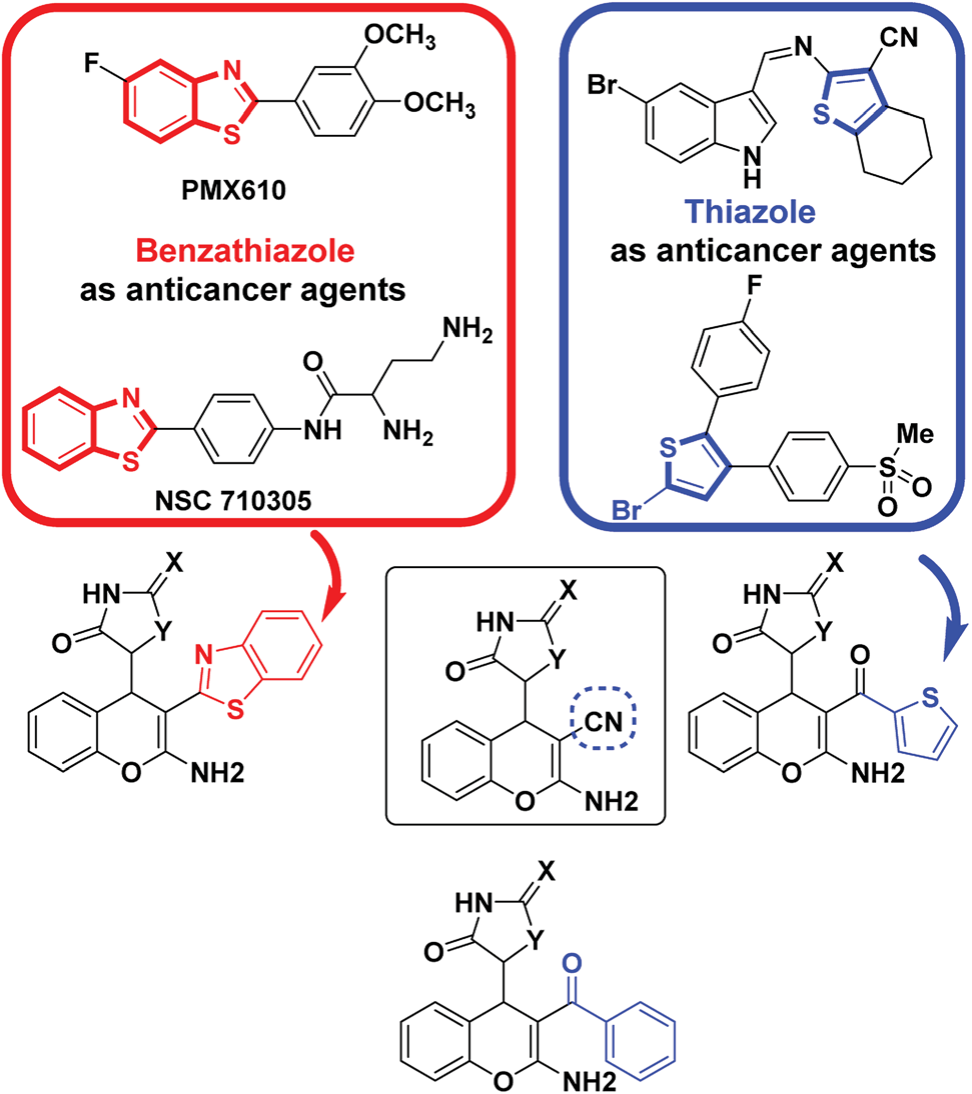
Design process based on the inspiration from bioactive heterocyclic scaffolds.

**Fig. 2:**
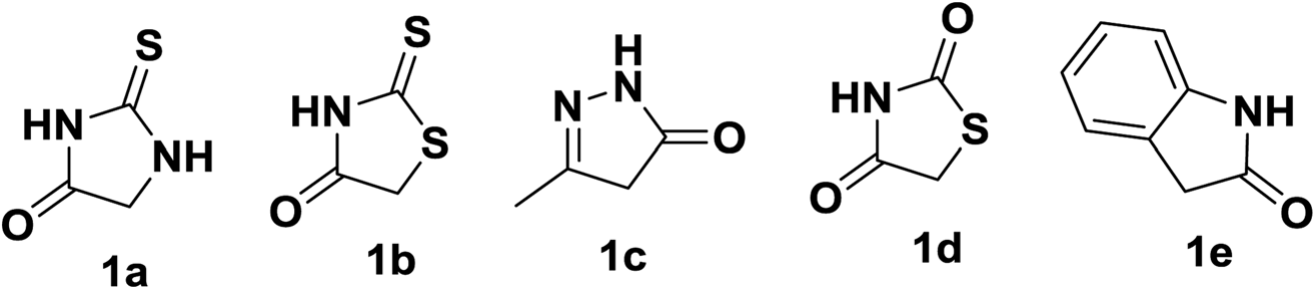
Heterocyclic carbon nucleophiles used in present work.

**Fig. 3:**
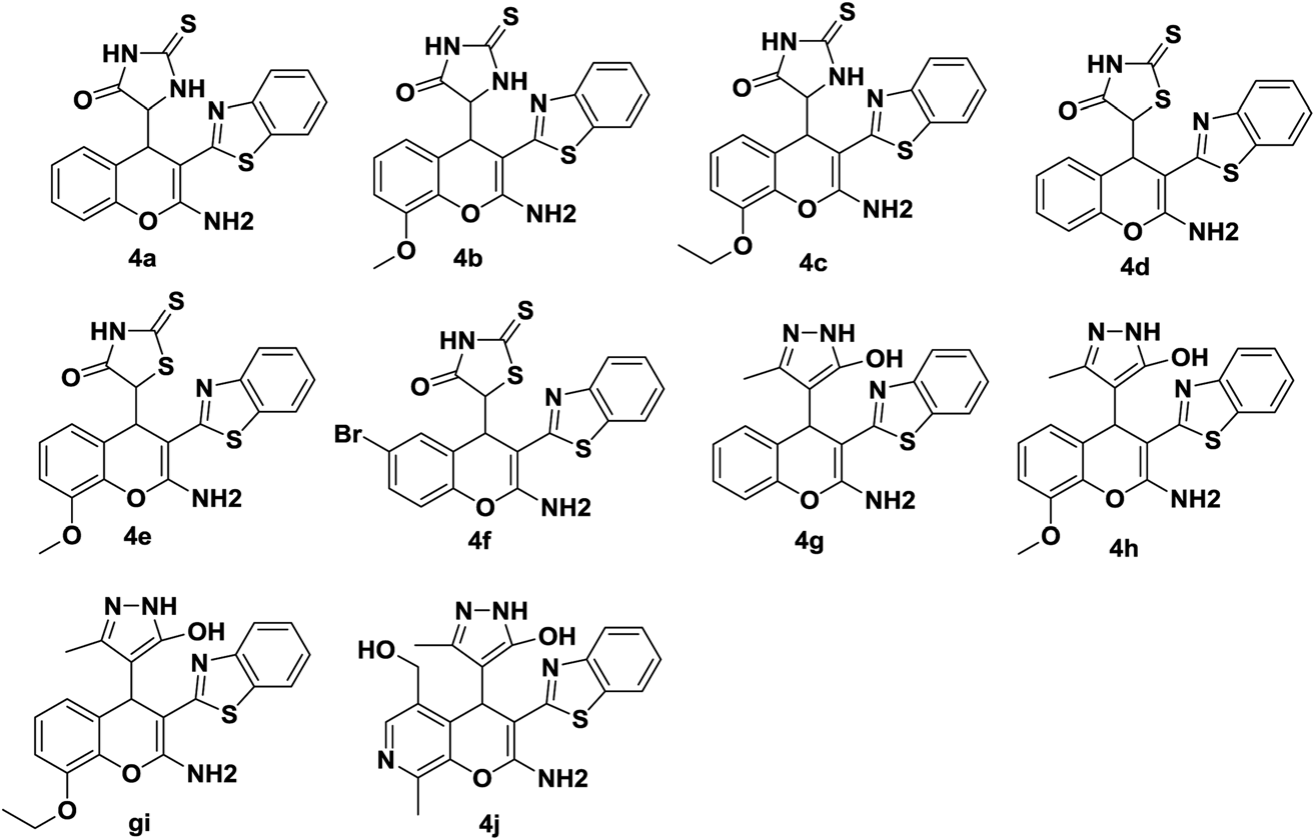
Substrate scope for the three component reaction (Scheme 1).

Our research group is actively engaged in developing multicomponent reaction protocols for accessing diverse scaffolds particularly 4-heterocycle-substituted-4*H*-chromene with inherent flexibility for incorporating appendages including replacing the benzene ring of chromene moiety by heterocyclic ring, by innovative design and synthesis of building blocks.^28–34^ Herein we disclose the design (Fig. 1) and synthesis of hybrid heterocyclic's by simultaneous incorporation of substituent's at 3 and 4 positions of 4*H*-chromenes in an efficient three component reaction (Fig. 2 and 3).

## Results and discussions

**Scheme 1:**
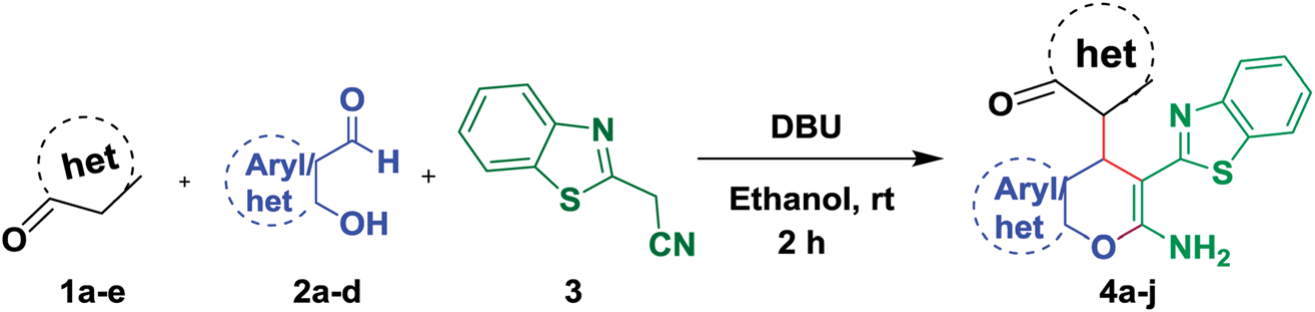
Three component reaction for the synthesis of 4a–j.

Initially we planned to investigate benzothiazole acetonitrile in the three component reaction with salicylaldehyde and heterocyclic carbon nucleophile as depicted in Scheme 1.

**Scheme 2:**
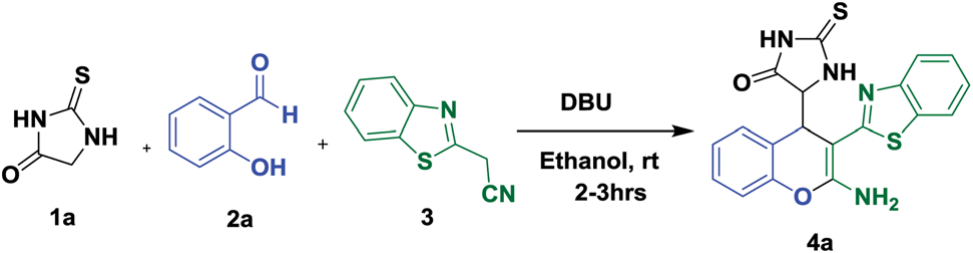
Three component reaction for the synthesis of 4a.

**Scheme 3:**
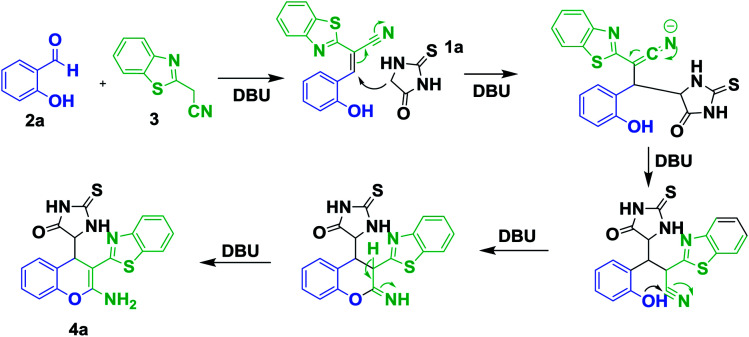
Plausible mechanism for the formation of 4a.

As a result the model reaction was performed between heterocyclic carbon nucleophile (1.0 equiv.) (1a), salicylaldehyde (1.0 equiv.) (2a), and benzothiazole acetonitrile (1.2 equiv.) (3) at room temperature to afford the required product in 95% yield in 2 h (Scheme 2). Then the reaction was examined with different bases (Table 1, entries 1–13). The required product was not formed when the reaction was conducted in the presence of acids. Under strong basic conditions, the reaction afforded trace amount of the expected product (4a) along with the condensed product of 2a and 3. Therefore the reaction was performed using mild bases, after screening various bases, 0.5 equiv. DBU was observed to be the best catalyst as it afforded the product in 2 h in excellent yield 95% (Table 1, entry 5). Later on increasing the base at 1.0 equiv. the resulting product was decreased. The structure of the product 4a was confirmed by ^1^H, ^13^C NMR and HRMS spectra (Scheme 2). A plausible reaction mechanism for 4a has been proposed and is illustrated in Scheme 3.

**Table 1:** Optimization of reaction condition for the synthesis of 4a

Entry	Solvent	Base	Equiv.	Time (h)	Yield (%)
1	MeOH	Piperidine	1.0	8	60
2	MeOH	DBU	0.5	3	85
3	MeOH	Et_3_N	1.0	6.5	70
4	EtOH	Et_3_N	1.0	5	80
**5**	**EtOH**	**DBU**	**0.5**	**2**	**95**
6	EtOH	DBU	1.0	2	85
7	EtOH	DABCO	1.0	4	75
8	EtOH	DMAP	1.0	4	60
9	EtOH	Piperidine	1.0	5	73
10	i-PrOH	Piperidine	1.0	12	55
11	i-PrOH	DBU	1.0	8	65
12	MeCN	DBU	1.0	12	50
13	H_2_O	DBU	1.0	12	—

**Scheme 4:**
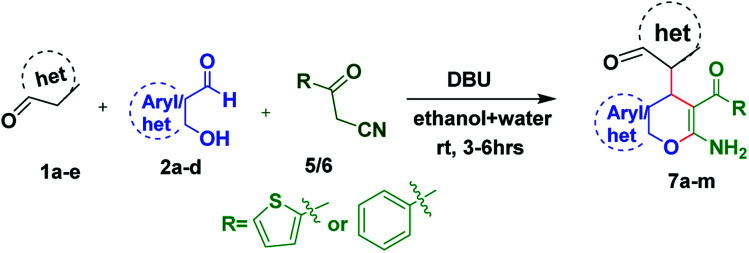
Three component reaction for the synthesis of 7a–m.

To extend the scope of this methodology, the three component reaction was further examined with thienoylacetonitrile as well as benzoylacetonitrile afford to heterocyclic carbon nucleophile and salicylaldehyde derivatives (Scheme 4).

**Scheme 5:**
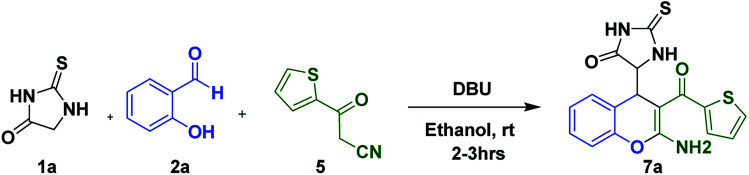
Three component reaction for the synthesis of 7a.

**Fig. 4:**
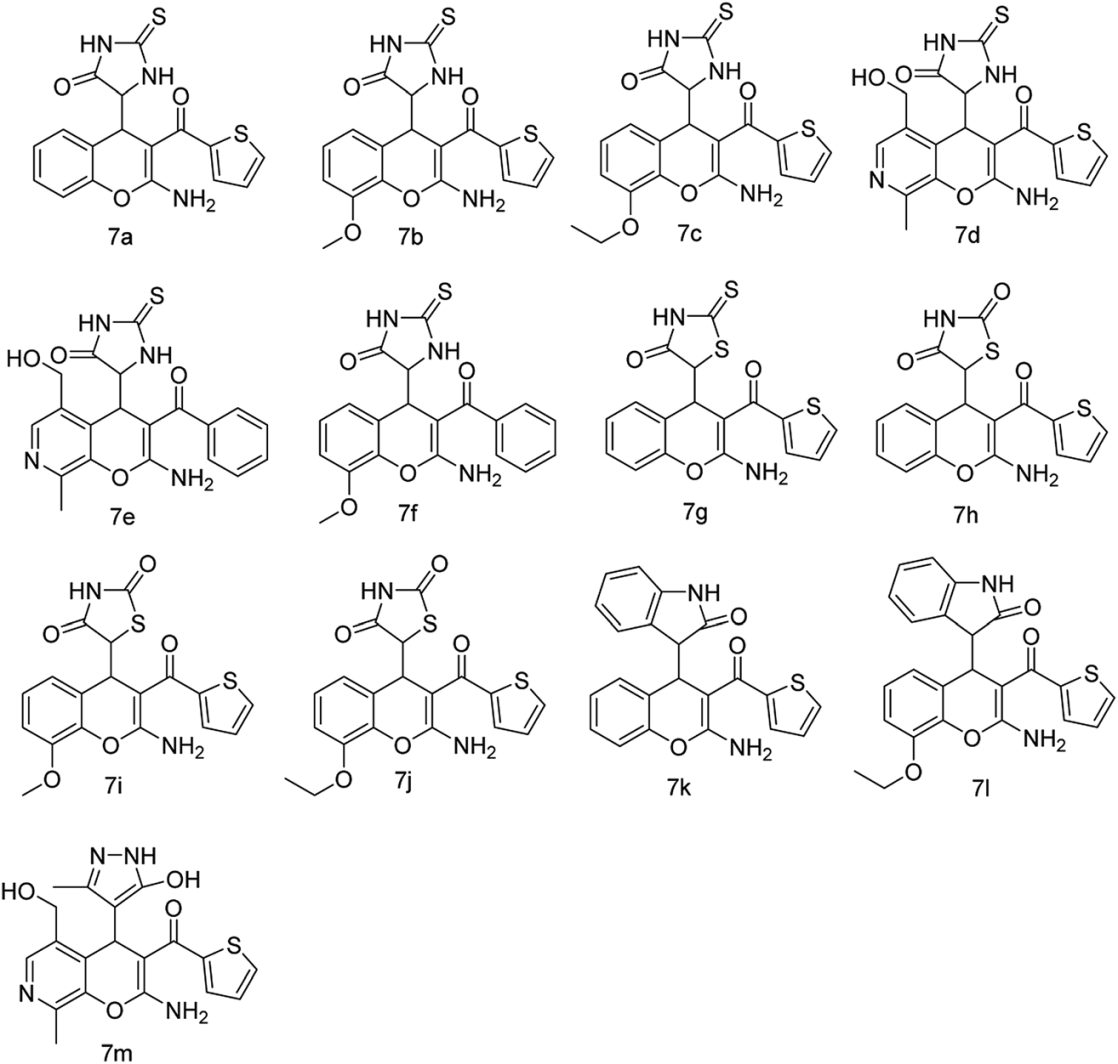
Substrate scope for the three component reaction (Scheme 4).

**Scheme 6:**
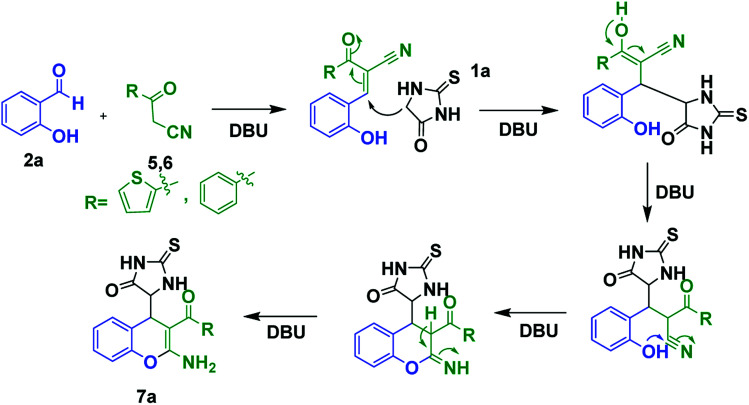
Plausible mechanism for the formation of 7a.

Accordingly a model reaction was performed with 2-thienoylacetonitrile (1.2 equiv.) (5) with 1a (1.0 equiv.) and 2a (1.0 equiv.) under the optimized reaction conditions. The reaction afforded the expected product (7a) in 83% yield (Scheme 5). Therefore, the reaction was conducted in various solvents in order to increase the yield (Table 2, entries 1–14). Among all the solvents, ethanol and water mixture with 0.5 equiv. DBU catalyst afforded the product in 3 h in excellent yield 92% (Table 2, entry 7). The structure of the compound 7a was confirmed by ^1^H, ^13^C NMR and HRMS spectra. Then the library of compounds 7a–m was synthesized and the results were summarized (Fig. 4, entries 11–23). A possible mechanism for the formation of 7a was given in Scheme 6.

**Table 2:** Optimization of reaction condition for the synthesis of 7a

Entry	Solvent	Base	Equiv.	Time (h)	Yield (%)
1	MeOH	DABCO	1.0	4	60
2	MeOH	Piperidine	0.5	3.5	77
3	MeOH	DBU	1.0	3	80
4	MeOH + H_2_O	DBU	1.0	3	85
5	EtOH	Piperidine	1.0	3.5	80
6	EtOH + H_2_O	DBU	1.0	3	86
**7**	**EtOH** + **H**_**2**_**O**	**DBU**	**0.5**	**3**	**92**
8	EtOH	DBU	1.0	3	83
9	EtOH	DABCO	1.0	5	72
10	i-PrOH	DBU	1.0	6	75
11	i-PrOH + H_2_O	DBU	1.0	8	70
12	i-PrOH	DABCO	1.0	6	55
13	MeCN	DMAP	1.0	8	40
14	MeCN	DBU	1.0	5	50

## Conclusion

We have designed and developed a facile one pot three component reaction protocol for the synthesis of 3,4-heterocyclic substituted 4*H*-chromenes. These benzothiazole/thiophene-thiazolidinedione-4*H*-chromene hybrids are expected to possess enhanced anticancer activity as the three bioactive moieties are embedded in a single molecule.

## Conflicts of interest

There are no conflicts of interest to declare.

## Supplementary Material

RA-008-C8RA08262F-s001
