# Supplementary material for: Design and synthesis of benzothiazole/thiophene-4H-chromene hybrids
Source: RSC Adv. 2018 Dec 13;8(73):41675–80. doi: 10.1039/c8ra08262f (PMC9091940; doi:10.1039/c8ra08262f)
Supplement: RA-008-C8RA08262F-s001 [file RA-008-C8RA08262F-s001.pdf]

Electronic Supplementary Material (ESI) for RSC Advances.
This journal is © The Royal Society of Chemistry 2018

Supplementary data

Design and Synthesis of benzothiazole/thiophene-4*H*-chromene hybrids

Pazhanivel Lakshmanan and Gnanasambandam Vasuki*

Department of Chemistry, Pondicherry University, RV Nagar, Kalapet Pondicherry-605014, India.

Corresponding author E-mail: vasukig@gmail.com.

Experimental section

General information: All chemicals were reagent grade purchased from Sigma Aldrich, Merck and Alfa Aesar used without purification. The reactions were monitored by thin layer chromatography (TLC) on pre-coated Aluminium plate of silica gel G/UV-254 of 0.25 mm thickness (Merck 60 F-254), spot were visualised under short UV light.NMR spectra were recorded on a BrukerAvance-II 400FT spectrometer at 400 MHz (1H) and 100(13C) IN DMSO using TMS as an internal reference. Mass spectra were recorded on a Water UPLC-TQD mass spectrometer.Melting points were determined by open glass capillary method and were uncorrected.

4. General procedure for synthesis of 5-(2-amino-3-(benzo[d]thiazol-2-yl)-4H-chromen-4-yl)-2-thioxoimidazolidin-4-one derivatives. To a stirred solution of ethanol were added a mixture of thiohydantoin (1.0equiv) ,benzothiazole acetonitrile (1.2equiv) and salicylaldehyde (1.0equiv) derivatives at room temperature with vigorous stirring for appropriate time (scheme 2). The precipitate was filtered, washed with ethanol 5ml x2 .The product were obtained uncontaminated by TLC and spectral techniques.

4a. 5-(2-amino-3-(benzo[d]thiazol-2-yl)-4H-chromen-4-yl)-2-thioxoimidazolidin-4-one:

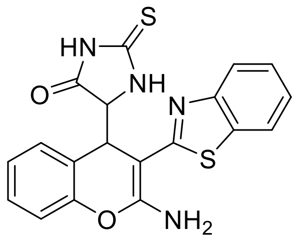

Pale yellow solid, yield 95%; m.p.=248-249°C; 1H NMR (400 MHz, DMSO-d_6): δ(ppm)=11.18(s,1H,NH), 10.37(s,1H,NH), 8.56(s,2H,NH_2), 7.96(d, J=7.6Hz, 1H), 7.78(d, J=8.0, Hz, 1H), 7.42-7.32(m,3H), 7.24-7.20(m, 1H),7.15-7.12(m,1H),7.07(d, J=8.4Hz, 1H), 4.40(d, J=3.2Hz, 1H), 4.17(d, J=2.8Hz, 1H); 13CNMR (DMSO-d6, 100 MHz)δ(ppm)= 182.4, 174.0, 167.9, 157.5, 153.4, 149.4, 131.0, 129.0, 128.7, 126.1, 123.8, 122.5, 121.4, 119.7, 118.6, 115.7, 74.2, 63.8;HR-MS m/z: calcd for C19H14N4O2S2 [M+H]+:395.0631 ; found:395.0634

4b. 5-(2-amino-3-(benzo[d]thiazol-2-yl)-8-methoxy-4H-chromen-4-yl)-2-thioxoimidazolidin-4-one

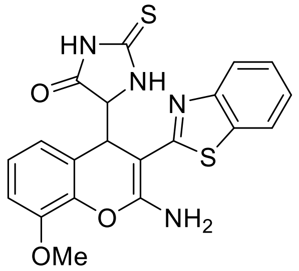

Yellow solid, yield 87%; m.p.=265-266°C; 1H NMR (400 MHz, DMSO-d_6): δ(ppm)=11.56(s, 1H,NH), 10.27(s,1H,NH), 8.57(s, 2H,NH_2),7.97(q, J=4.0,7.2Hz, 1H), 7.79(d, J=8.0Hz, 1H), 7.42-7.38(m, 1H), 7.24-7.20(m,1H), 7.08-7.03(m, 2H), 6.69(q, J=2.0, 4.8Hz, 1H),4.54(d, J=2Hz, 1H), 4.28(d, j=2.0Hz, 1H), 3.84(s,3H);13C NMR (DMSO-d6, 100 MHz)δ(ppm)=183.2, 175.0, 167.6, 157.2, 153.5, 147.0, 139.7, 131.0, 126.1, 124.0, 122.6, 121.5, 119.7, 119.7, 119.2, 111.8, 75.6, 65.9, 55.7, 48.6, 40.6.HR-MS m/z: calcd forC20H16N4O3S2 [M+H]+:425.0737; found:425.0730.

4c. 5-(2-amino-3-(benzo[d]thiazol-2-yl)-8-ethoxy-4H-chromen-4-yl)-2-thioxoimidazolidin-4-one

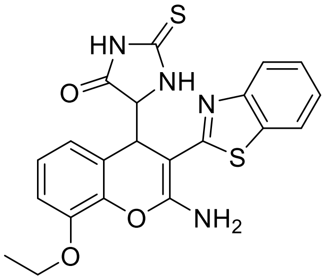

Pale yellow solid, yield 83%; m.p.=256-257°C; 1H NMR (400 MHz, DMSO-d_6): δ(ppm)=11.55(s,1H,NH), 10.28(s,1H,NH), 8.52(s,2H, NH_2), 7.97(d, J=7.6Hz, 1H), 7.79(d, J=8.0Hz, 1H), 7.42-7.38(m,1H), 7.24-7.20(m, 1H), 7.05-7.00(m, 2H), 6.68(q, J=2.8,3.6Hz, 1H), 4.54(d, J=1.6Hz, 1H), 4.28(d, J=2.0Hz, 1H), 4.11(q, J=6.8,3.6Hz, 2H), 1.37(t, J=6.8,6.8Hz, 3H);13C NMR (DMSO-d6, 100 MHz)δ(ppm)=183.1, 175.0, 1167.6, 157.2, 153.5, 146.1, 140.0, 131.0, 126.1, 124.0, 122.6, 121.5, 119.7, 119.7, 119.2, 113.0, 75.7, 66.0, 64.2, 40.7, 14.7.HR-MS m/z: calcd for C21H18N4O3S2 [M+H]+:439.0893;found:439.0899.

4d. 5-(2-amino-3-(benzo[d]thiazol-2-yl)-4H-chromen-4-yl)-2-thioxothiazolidin-4-one

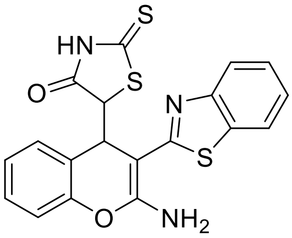

Yellow solid, yield 88%; m.p.=240-241°C; 1H NMR (400 MHz, DMSO-d_6): δ(ppm)=8.42(s,1H,NH_2), 7.98(d, J=7.6Hz, 1H), 7.77(d, J=8.0Hz, 1H), 7.41-737(m, 1H), 7.35-7.27(m, 1H), 7.24(t, J=0.8,7.2Hz, 1H), 7.21-7.17(m, 1H), 7.11-7.06(m, 2H), 4.81(d, J=3.2Hz, 1H), 4.61(d, J=2.8Hz, 1H);13C NMR (DMSO-d6, 100 MHz)δ(ppm)=211.8, 188.9, 167.8, 156.5, 153.3, 150.4, 131.0, 128.6, 126.0, 124.0, 122.6, 1215, 120.4, 119.8, 115.4, 78.5, 66.2, 44.3.HR-MS m/z: calcd for C19H13N3O2S3 [M+H]+:412.0243; found:412.0243.

4e. 5-(2-amino-3-(benzo[d]thiazol-2-yl)-8-methoxy-4H-chromen-4-yl)-2-thioxothiazolidin-4-one

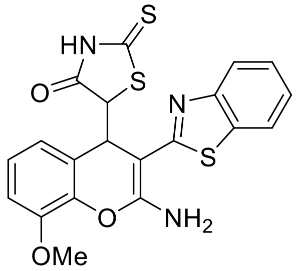

Yellow solid, yield 85%; m.p.=253-254°C; 1H NMR (400 MHz, DMSO-d_6): δ(ppm)=13.17(s,1H,NH), 8.59(s,2H,NH_2), 7.99(d, J=7.6Hz,1H), 7.80(d, J=8.0Hz, 1H), 7.43-7.39(m,1H), 7.27-7.22(m, 1H), 7.13-7.06(m, 2H),6.73(q, J=1.6,5.6Hz, 1H),5.28(d, J=3.2Hz, 1H), 4.70(d, J=2.8Hz, 1H), 3.85(s, 3H); 13C NMR (DMSO-d6, 100 MHz)δ(ppm)=176.9, 167.2, 156.7, 153.3, 147.0, 139.7, 131.5, 126.7, 124.4, 122.9, 121.6, 120.0, 119.9, 119.2, 112.1, 76.5, 63.0, 55.7, 48.6, 40.5. HR-MS m/z: calcdfor C20H15N3O3S3[M+H]+:442.0348 ; found:442.0348.

4f. 5-(2-amino-3-(benzo[d]thiazol-2-yl)-6-bromo-4H-chromen-4-yl)-2-thioxothiazolidin-4-one

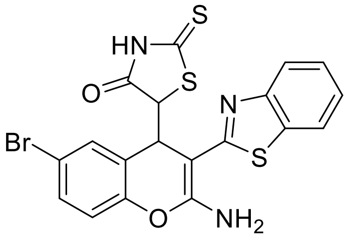

Yellow solid, yield 70%; m.p.=272-273°C; 1H NMR (400 MHz, DMSO-d_6): δ(ppm)=8.43(s,2H,NH_2), 7.98(d, J=7.6Hz, 1H), 7.78(d, J=7.6Hz,1H), 7.80-7.40(m, 1H), 7.38(d, J=1.2Hz, 1H), 7.30(d, J=2.4Hz, 1H), 7.25-7.21(m, 1H), 7.06(d, J=8.8Hz, 1H), 4.82(d, J=3.2Hz, 1H), 4.58(d, J=2.8Hz, 1H); 13C NMR (DMSO-d6, 100 MHz)δ(ppm)=211.8, 189.1, 167.5, 156.1, 153.2, 149.8, 131.3, 131.1, 131.0, 126.1, 122.9, 122.7, 121.5, 119.9, 117.7, 115.4, 78.0, 66.3,HR-MS m/z: calcd for C19H12BrN3O2S3
[M+H]+: 489.9348; found:489.9344.

4g. 4-(2-amino-3-(benzo[d]thiazol-2-yl)-4H-chromen-4-yl)-3-methyl-1H-pyrazol-5-ol

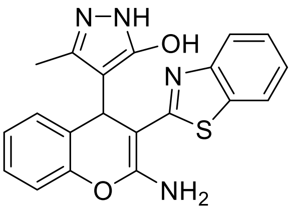

White solid, yield 93%; m.p.=245-246°C; 1H NMR (400 MHz, DMSO-d_6): δ(ppm)=10.47(s, 2H, NH,OH), 8.37(s, 2H, NH_2), 7.87(d, J=8.0Hz, 1H), 7.74(d, J=8.4Hz, 1H), 7.34(t, J=7.2, 7.6Hz,1H), 7.29(d, J=7.6Hz, 1H), 7.22-7.14(m, 2H), 7.07(q, J=7.2, 6.4Hz, 2H), 4.88(s, 1H), 2.04(s, 3H);13C NMR (DMSO-d6, 100 MHz)δ(ppm)=169.2, 159.4, 155.5, 152.9, 147.9, 136.1,131.7, 129.9, 127.4, 125.7, 125.4, 124.2, 122.3, 121.1, 119.6, 115.4, 105.3, 78.14, 31.0, 10.1HR-MS m/z: calcd for C20H16N4O2S
[M+H]+: 377.1067; found:377.1069.

4h. 4-(2-amino-3-(benzo[d]thiazol-2-yl)-8-methoxy-4H-chromen-4-yl)-3-methyl-1H-pyrazol-5-ol

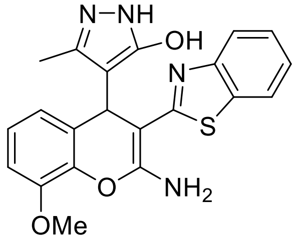

Pink solid, yield 83%; m.p.=263-264°C; 1H NMR (400 MHz, DMSO-d_6): δ(ppm)=10.45(s, 2H, NH,OH), 8.41(s, 2H, NH_2), 7.86(d, J=8 Hz, 1H), 7.74(d, J=8Hz, 1H), 7.34(t, J=7.6,7.6Hz, 1H), 7.15(t, J=7.6,7.6Hz,1H), 7.00(t, J=8.0,7.6Hz, 1H), 6.87(q, J=8.4,5.2Hz, 2H), 4.86(s,1H), 3.83(s,3H), 2.03(s, 3H);13C NMR (DMSO-d6, 100 MHz)δ(ppm)=169.2, 159.4, 155.5, 152.9, 146.7, 137.3, 136.1, 131.7, 126.1, 125.7, 123.8, 122.2, 121.1, 120.3, 119.6, 109.8, 105.2, 78.0, 55.6, 31.0, 10.1.HR-MS m/z: calcd forC21H18N4O3S[M+H]+:407.1172; found:407.1181.

4i. 4-(2-amino-3-(benzo[d]thiazol-2-yl)-8-ethoxy-4H-chromen-4-yl)-3-methyl-1H-pyrazol-5-ol

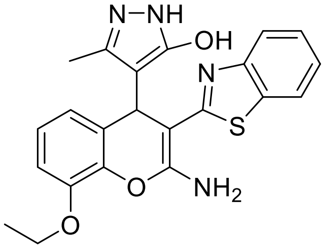

Pink solid, yield 78%; m.p.=218-219°C; 1H NMR (400 MHz, DMSO-d_6): δ(ppm)=10.44(s, 2H, NH,OH), 8.37(s,2H,NH_2), 7.86(d, J=7.6Hz, 1H), 7.74(d, J=8.0Hz, 1H), 7.36-7.32(m,1H), 7.18-7.14(m,1H), 6.97(t, J=10.8,4.8Hz, 1H), 6.85(t, J=9.6,8.4Hz, 2H), 4.85(s,1H), 4.10(q, J=7.2,6.8Hz,2H), 2.03(s,3H), 1.38(t, J=7.2,6.8Hz,3H);13C NMR (DMSO-d6, 100 MHz)δ(ppm)=169.2, 159.4, 155.5, 152.9, 145.9, 137.6, 136.1, 131.7, 126.2, 125.7, 13.8, 122.2, 121.1, 120.3, 119.5, 111.0, 105.3, 78.0, 64.0, 31.1, 14.7, 10.1.HR-MS m/z: calcd for C22H20N4O3S [M+H]+:421.1329 ; found:421.1334.

4j. 4-(2-amino-3-(benzo[d]thiazol-2-yl)-5-(hydroxymethyl)-8-methyl-4H-pyrano[2,3-c]pyridin-4-yl)-3-methyl-1H-pyrazol-5-ol

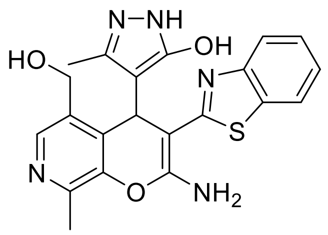

Pale yellow solid, yield 91%; m.p.=260-261°C; 1H NMR (400 MHz, DMSO-d_6): δ(ppm)=10.97(s, 1H,NH), 9.87(s, 1H, OH), 8.35(s, 2H, NH_2), 8.16(s, 1H), 7.89(d, J=8.0, 1H), 7.74(d, J=8.0Hz,1H), 7.35(t, J=7.6,7.6Hz, 1H), 7.18(t, J=7.6,7.2Hz, 1H), 5.31(s, 1H, OH), 4.91(s, 1H), 4.70(d, J=14Hz, 1H), 4.39(d, J=14Hz, 1H), 2.53(s, 3H),2.15(s, 3H);13C NMR (DMSO-d6, 100 MHz)δ(ppm)=169.0, 159.3, 155.3, 152.9, 144.2, 143.5, 142.3, 136.7, 132.6, 131.5, 129.1, 125.8, 122.4, 121.1, 119.6, 130.0, 78.8, 58.0, 27.8, 18.7 , 10.2.HR-MS m/z: calcd forC21H19N5O3S [M+H]+: 422.1281; found:422.1289.

7. General procedure for synthesis of 5-(2-amino-3-(thiophene-2-carbonyl)-4H-chromen-4-yl)-2-thioxoimidazolidin-4-one derivatives. To a stirred solution of ethanol and water (1:1) ratio were added a mixture of 2-thenoyl acetonitrile(3-oxo-3-(thiophen-2-yl)propanenitrile) (1.2 equiv), thiohydantoin(1.0 equiv) and salicylaldehyde(1.0 equiv) derivatives at ambient temperature with vigorous stirring for appropriate time (scheme 5). The precipitate was filtered, washed with ethanol and water (1:1) ratio10x3. The product were obtained, uncontaminated by TLC and spectral techniques.

7a. 5-(2-amino-3-(thiophene-2-carbonyl)-4H-chromen-4-yl)-2-thioxoimidazolidin-4-one

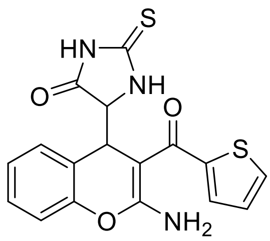

White solid, yield 92%; m.p.=233-234°C; 1H NMR (400 MHz, DMSO-d_6): δ(ppm)=11.49(s,1H,NH), 10.21(s,1H,NH), 9.37(s,2H,NH2), 7.78(d, J=4.8Hz,1H), 7.72(d, J=3.2Hz, 1H), 7.34-7.30(m,1H), 7.20(t, J=4.0,4.4Hz, 1H), 7.14-7.07(m, 3H), 4.90(d, J=0.8Hz,1H); 13C NMR (DMSO-d6, 100 MHz)δ(ppm)=183.1, 180.3, 174.8, 165.0, 150.2, 146.2, 130.4, 1290, 128.2, 128.0, 127.9, 124.4, 120.0, 115.6, 83.90, 68.33, 37.95, HR-MS m/z: calcd for $C_{17}H_{13}N_3O_3S_2$ [M+H]+: 372.0471; found: 372.048.

7b. 5-(2-amino-8-methoxy-3-(thiophene-2-carbonyl)-4H-chromen-4-yl)-2-thioxoimidazolidin-4-one

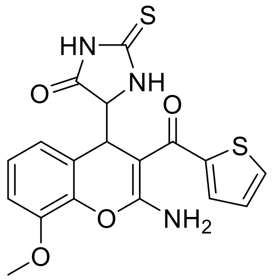

Yellow solid, yield 90%; m.p.=268-269°C; 1H NMR (400 MHz, DMSO-d_6): δ(ppm)=11.50(s,1H,NH), 10.21(s,1H,NH), 9.42(s,2H,NH_2), 7.77(d, J=4.4Hz,1H), 7.70(d, J=9.6Hz,1H), 7.20(t, J=4.4,4.0Hz,1H), 7.04(d, J=6.0Hz,2H), 6.65(d, J=5.6Hz,1H), 4.89(d, J=7.2Hz, 1H), 4.32(d, J=8.0Hz, 1H), 3.83(s,3H); 13C NMR (DMSO-d6, 100 MHz)δ(ppm)=183.1, 180.3, 174.8, 164.0, 146.8, 146.2, 139.4, 130.5, 128.0, 127.9, 124.3, 121.0, 119.4, 111.9, 83.7, 68.3, 55.9, 37.8. : HR-MS m/z: calcd for C18H15N3O4S2 [M+H]+: 402.0577; found: 402.0584.

7c. 5-(2-amino-8-ethoxy-3-(thiophene-2-carbonyl)-4H-chromen-4-yl)-2-thioxoimidazolidin-4-one

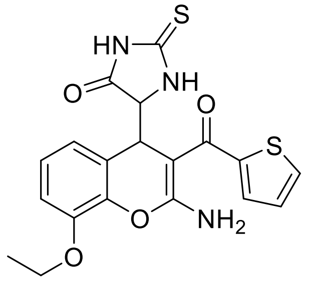

Yellow solid, yield 80%; m.p=262-263°C; 1H NMR (400 MHz, DMSO-d_6): δ(ppm)=11.49(s,1H,NH), 10.22(s,1H,NH), 9.41(s,2H,NH_2), 7.77(d, J=4.8Hz, 1H), 7.71(d, J=3.6Hz, 1H), 7.19(t, J=4.4,4.4Hz, 1H), 7.01(t, J=2.4,3.2Hz, 2H), 6.64(q, J=3.2,2.4Hz, 1H), 4.89(d, J=1.6Hz, 1H), 4.32(s, 1H), 4.10(q, J=7.2,6.8Hz, 2H), 1.35(t, J=6.8Hz, 3H); 13C NMR (DMSO-d6, 100 MHz)δ(ppm)=183.1, 180.2, 174.8, 164.8, 146.3, 146.0, 139.7, 130.5, 128.0, 127.9, 124.3, 121.0, 119.4, 113.3, 83.8, 68.4, 64.3, 37.9, 25.5, 14.6. HR-MS m/z: calcdfor C19H17N3O4S2 [M+H]+: 416.0733; found: 416.0743.

7d. 5-(2-amino-5-(hydroxymethyl)-8-methyl-3-(thiophene-2-carbonyl)-4H-pyrano[2,3-c]pyridin-4-yl)-2-thioxoimidazolidin-4-one

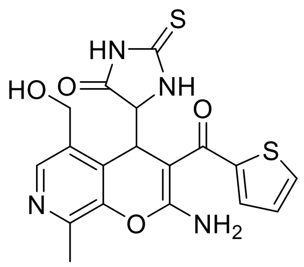

Pink solid, yield 89%; m.p=259-260°C; 1H NMR (400 MHz, DMSO-d_6): δ(ppm)=11.50(s,1H,NH), 9.86(s,1H,NH), 8.87(s,2H,NH_2), 8.25(s,1H), 7.69(t, J=4.4,0.8Hz,1H), 7.52(d, J=3.2Hz,1H), 7.06(q, J=4.0,0.8Hz, 1H), 5.46(d, J=4.8Hz,1H), 5.25(s, 1H), 4.66(d, J=4.8Hz, 1H), 4.61(d,J=4.0Hz, 1H0, 4.36(d, J=0.8Hz, 1H), 2.51(s,3H); 13C NMR (DMSO-d6, 100 MHz)δ(ppm)=183.4, 181.9, 173.5, 164.8, 145.8, 145.7, 145.4, 143.9, 131.4, 129.9, 129.8, 128.4, 127.2, 79.6, 66.6, 58.6, 34.8, 18.6. HR-MS m/z: calcd for C18H16N4O4S2 [M+H]+: 417.0686; found: 417.0695.

7e. 5-(2-amino-3-benzoyl-5-(hydroxymethyl)-8-methyl-4H-pyrano[2,3-c]pyridin-4-yl)-2-thioxoimidazolidin-4-one

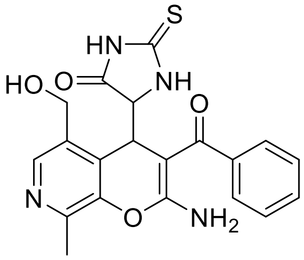

Pale yellow solid, yield 91%; m.p=238-239°C; 1H NMR (400 MHz, DMSO-d_6): δ(ppm)=11.50(s,1H,NH), 9.76(s,1H,NH), 8.80(s, 2H,NH_2), 8.23(s, 1H), 7.37-7.30(m, 5H), 5.38(t, J=4.8,4.4Hz, 1H), 4.72(d, J=2.8Hz, 1H), 4.52(d, J=36Hz, 2H), 4.29(s, 1H),2.52(s, 3H); 13C NMR (DMSO-d6, 100 MHz)δ(ppm)= 191.9, 183.4, 173.4, 164.8, 146.0, 145.9, 143.7, 140.5, 131.2, 130.3, 129.6, 127.9, 127.8, 79.5, 66.5, 58.5, 35.5, 18.7. HR-MS m/z: calcd for C20H18N4O4S [M+H]+: 411.1122; found: 411.1119.

7f. 5-(2-amino-3-benzoyl-8-methoxy-4H-chromen-4-yl)-2-thioxoimidazolidin-4-one

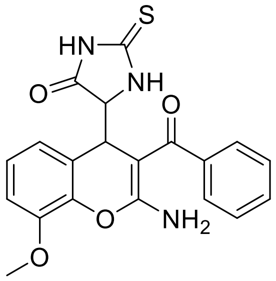

Yellow solid, yield 83%; m.p=214-215°C; 1H NMR (400 MHz, DMSO-d_6): δ(ppm)=11.35(s,1H,NH), 9.95(s, 1H,NH), 9.13(s, 2H,NH_2), 7.45-7.42(m, 3H), 7.36(q, J=3.6,3.6Hz,2H), 7.02(t, J=0.8,4.4Hz,2H), 6.54(t, J=4.4,4.8Hz, 1H),4.29(d, J=2.8Hz, 1H), 3.9(d, J=1.6Hz, 1H), 3.83(s,3H); 13C NMR (DMSO-d6, 100 MHz)δ(ppm)=192.1, 183.0, 174.1, 164.1, 146.9, 141.5, 139.7, 129.0, 128.4, 126.2, 124.2, 121.4, 119.5, 111.8, 82.7, 68.4, 55.8, 38.4. HR-MS m/z: calcd for C20H17N3O4S [M+H]+: 396.1013; found: 396.1021.

7g. 5-(2-amino-3-(thiophene-2-carbonyl)-4H-chromen-4-yl)-2-thioxothiazolidin-4-one

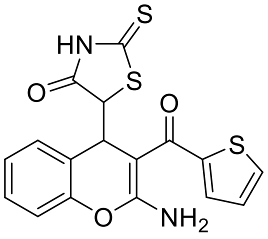

Yellow solid, yield 86%; m.p=208-209°C; 1H NMR (400 MHz, DMSO-d_6): δ(ppm)=13.07(s,1H,NH), 9.28(s, 2H,NH_2), 7.81(t, J=5.2, 4.4Hz, 2H), 7.38-7.35(m, 1H), 7.23-7.13(m, 4H)5.29(d, J=3.2Hz, 1H), 4.95(d, J=3.2Hz, 1H); 13C NMR (DMSO-d6, 100 MHz)δ(ppm)=203.1, 180.9, 176.6, 164.1, 150.0, 145.5, 130.8, 129.4, 128.4, 128.2, 128.0, 124.7, 120.2, 115.8, 84.1, 65.1,38.0 HR-MS m/z: calcd for. C17H12N2O3S3 [M+H]+: 389.0083; found: 389.0090.

7h. 5-(2-amino-3-(thiophene-2-carbonyl)-4H-chromen-4-yl)thiazolidine-2,4-dione

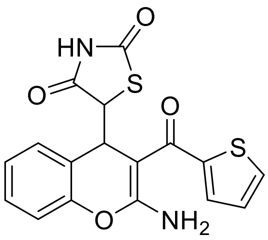

White solid, yield 78%; m.p=223-224°C; 1H NMR (400 MHz, DMSO-d_6): δ(ppm)=12.03(s, 1H, NH), 9.30(s, 2H, NH_2), 7.83(q, J=3.2,8.8Hz, 2H), 7.40-7.36(m, 1H), 7.23-7.14(m,4H), 5.31(d, J=2.8Hz,1H), 4.82(d, J=2.8Hz,1H); 13C NMR (DMSO-d6, 100 MHz)δ(ppm)=180.8, 174.4, 171.5, 164.2, 150.2, 145.7, 130.9, 129.4, 128.4, 128.3, 128.1, 124.7, 120.0, 115.8, 84.2, 62.1, 37.8. HR-MS m/z: calcd for C17H12N2O4S2 [M+H]+: 373.0311; found: 373.0322.

7i. 5-(2-amino-8-methoxy-3-(thiophene-2-carbonyl)-4H-chromen-4-yl)thiazolidine-2,4-dione

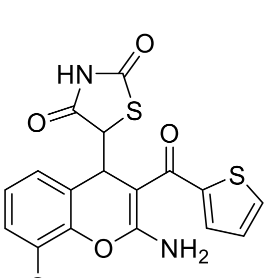

Yellow solid, yield 72%; m.p=249-250°C; 1H NMR (400 MHz, DMSO-d_6): δ(ppm)=12.02(s,1H, NH), 9.36(s,2H, NH_2), 7.84-7.81(m, 2H), 7.22(q, J=4.0,5.2Hz, 1H), 7.13-7.06(m, 2H), 6.70(q, J=2.0, 5.2Hz, 1H), 5.30(d, J=3.2Hz, 1H), 4.82(d, J=3.2Hz, 1H); 13C NMR (DMSO-d6, 100 MHz)δ(ppm)=180.8, 174.3, 171.5, 164.1, 146.9, 145.6, 139.3,

130.8, 128.3, 128.1, 124.6, 121.0, 119.4, 112.1, 84.1, 62.0, 55.7, 37.9. HR-MS m/z: calcd for C18H14N2O5S2 [M+H]+: 403.0417; found: 403.0426.

7j. 5-(2-amino-8-ethoxy-3-(thiophene-2-carbonyl)-4H-chromen-4-yl)thiazolidine-2,4-dione

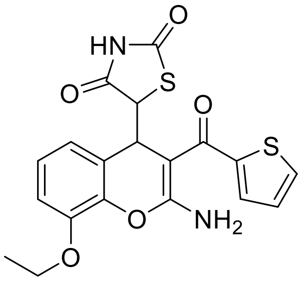

Yellow solid, yield 70%; m.p=236-237°C; 1H NMR (400 MHz, DMSO-d_6): δ(ppm)=12.02(s,1H,NH), 9.35(s,2H,NH_2), 7.83(q, J=3.6,4.8Hz, 2H), 7.22(q, J=4.0,1.2Hz, 1H), 7.07(t, J=4.4,2.0Hz, 2H), 6.69(q, J=2.8,3.6Hz,1H), 5.29(d, J=2.8Hz, 1H), 4.82(d, J=3.2Hz, 1H), 4.13(q, J=7.2,3.6Hz, 2H), (t, J=6.8,6.8Hz, 3H); 13C NMR (DMSO-d6, 100 MHz)δ(ppm)=180.0, 174.4, 171.6, 164.1, 146.1, 145.7, 139.6, 130.9, 128.3, 128.1, 124.6, 121.1, 119.5, 113.3, 84.2, 64.3, 62.1, 37.9, 14.6. HR-MS m/z: calcd for C19H16N2O5S2 [M+H]+: 417.0573; found: 417.0555.

7k. 3-(2-amino-3-(thiophene-2-carbonyl)-4H-chromen-4-yl)indolin-2-one

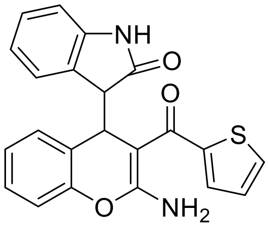

Pale yellow solid, yield 80%; m.p=256-257°C; 1H NMR (400 MHz, DMSO-d_6): δ(ppm)=10.41(s,1H,NH), 9.45(s, 2H,NH_2), 8.08(d, J=3.6Hz, 1H), 7.81(q, J=0.4,4.4Hz, 1H), 7.24(q, J=3.6,1.2Hz, 1H), 7.20(q, J=1.6,6.4Hz, 1H), 7.10-7.06(m,2H), 7.02-6.97(m,2H), 6.82-6.77(m,2H), 6.52(d, J=8.0Hz, 1H), 5.14(d, J=3.2Hz,1H), 3.67(d, J=3.2Hz, 1H); 13C NMR (DMSO-d6, 100 MHz)δ(ppm)=180.6, 176.9, 164.6, 148.9, 146.9, 142.8, 131.0, 128.4, 128.4, 127.9, 126.5, 124.1, 123.8, 121.5, 121.0, 115.1, 109.0, 85.1, 62.0, 54, 37.0, 25.5. HR-MS m/z: calcd for C22H16N2O3S [M+H]+: 389.0954; found: 388.0935.

7l. 3-(2-amino-8-ethoxy-3-(thiophene-2-carbonyl)-4H-chromen-4-yl)indolin-2-one

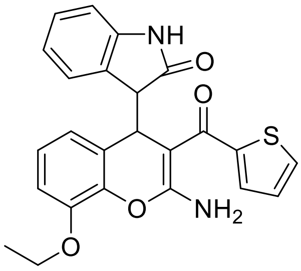

Yellow solid, yield 74%; m.p=271-272°C; 1H NMR (400 MHz, DMSO-d_6): δ(ppm)=10.40(s,1H, NH),9.51(s,2H,NH_2),8.07(d, J=3.2Hz, 1H), 7.81(q, J=0.8,4.4Hz, 1H), 7.23(q, J=4.0,1.2Hz, 1H), 7.06(d, J=7.6Hz, 1H), 7.00(t, J=7.6,7.6Hz, 1H), 6.90(t, J=8.0,7.6Hz, 1H), 6.81-6.75(m,3H), 6.55(d, J=8.0Hz, 1H), 5.13(d, J=6.8Hz, 1H), 3.93(q, J=6.8,10.4Hz, 2H), 3.66(d, J=28Hz, 1H), 1.20(t, J=6.8,68Hz, 3H); 13C NMR (DMSO-d6, 100 MHz)δ(ppm)=180.6, 176.8, 164.5, 146.9, 145.5, 142.8, 138.7, 131.0, 128.4, 128.3, 127.9, 126.5, 124.0, 123.8, 122.7, 121.0, 119.6, 113.1, 109.0, 85.0, 64.3, 54.1, 37.0, 14.5. HR-MS m/z: calcd for C24H20N2O4S [M+H]+: 433.1217; found: 433.1225.

7m. (2-amino-4-(5-hydroxy-3-methyl-1H-pyrazol-4-yl)-5-(hydroxymethyl)-8-methyl-4H-pyrano[2,3-c]pyridin-3-yl)(thiophen-2-yl)methanone

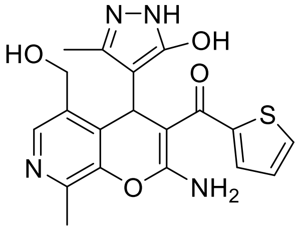

Pink solid, yield 84%; m.p=235-236°C; 1H NMR (400 MHz, DMSO-d_6): δ(ppm)=10.92(s,1H,OH), 9.46(s, 1H,NH), 8.90(s, 2H,NH_2), 8.11(s,1H), 7.71(d, J=4.8Hz, 1H), 7.59(d, J=3.6Hz, 1H), 7.12(t, J=4.4, 4.0Hz, 1H), 5.54(s,1H), 5.29(s, 1H), 4.48(q, J=5.2,8.0Hz, 1H), 4.30(d, J=11.6Hz, 1H), 1.52(s,3H); 13C NMR (DMSO-d6, 100 MHz)δ(ppm)=183.5, 163.2, 144.9, 144.3, 144.2, 142.6, 132.2, 131.1, 129.0, 127.8, 127.2, 104.6, 86.4, 58.1, 56.0, 25.9, 18.5,18.5, 8.7. HR-MS m/z: calcd for C19H18N4O4S [M+H]+: 399.1122; found: 399.1132.

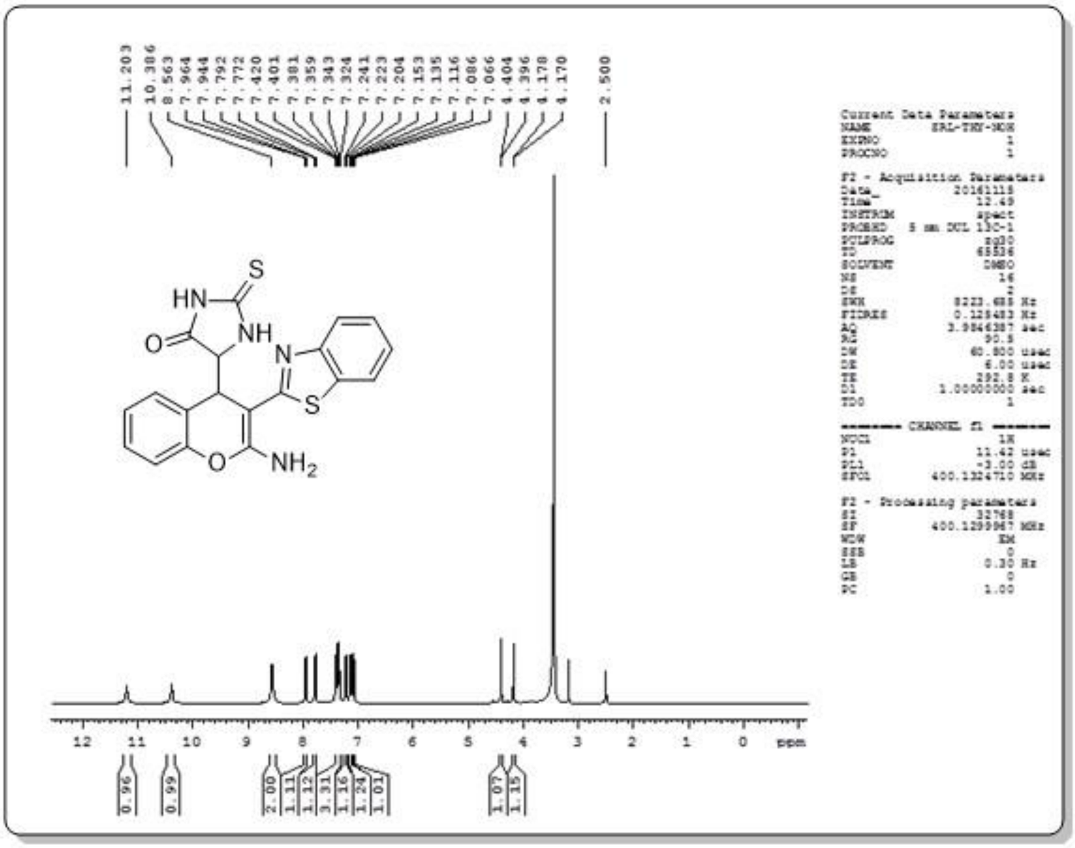

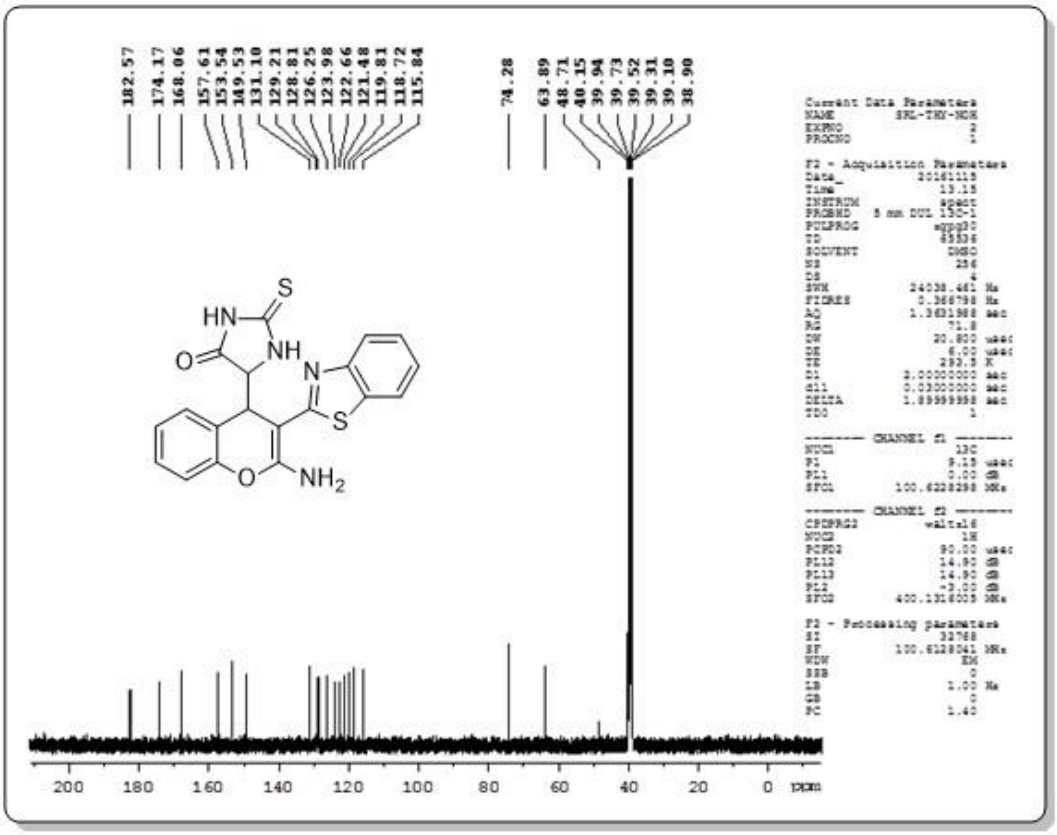

Fig 5: ^{1}H, ^{13}C spectrum of 5-(2-amino-3-(benzo[d]thiazol-2-yl)-4H-chromen-4-yl)-2-thioxoimidazolidin-4-one **4a**

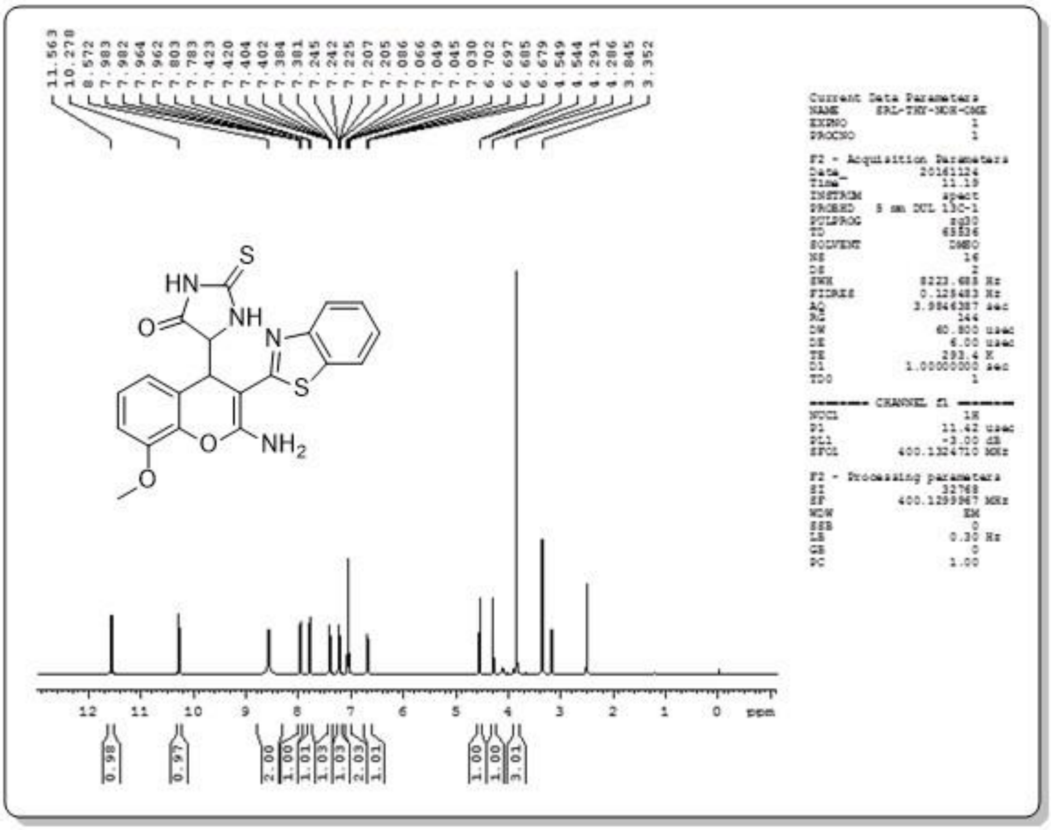

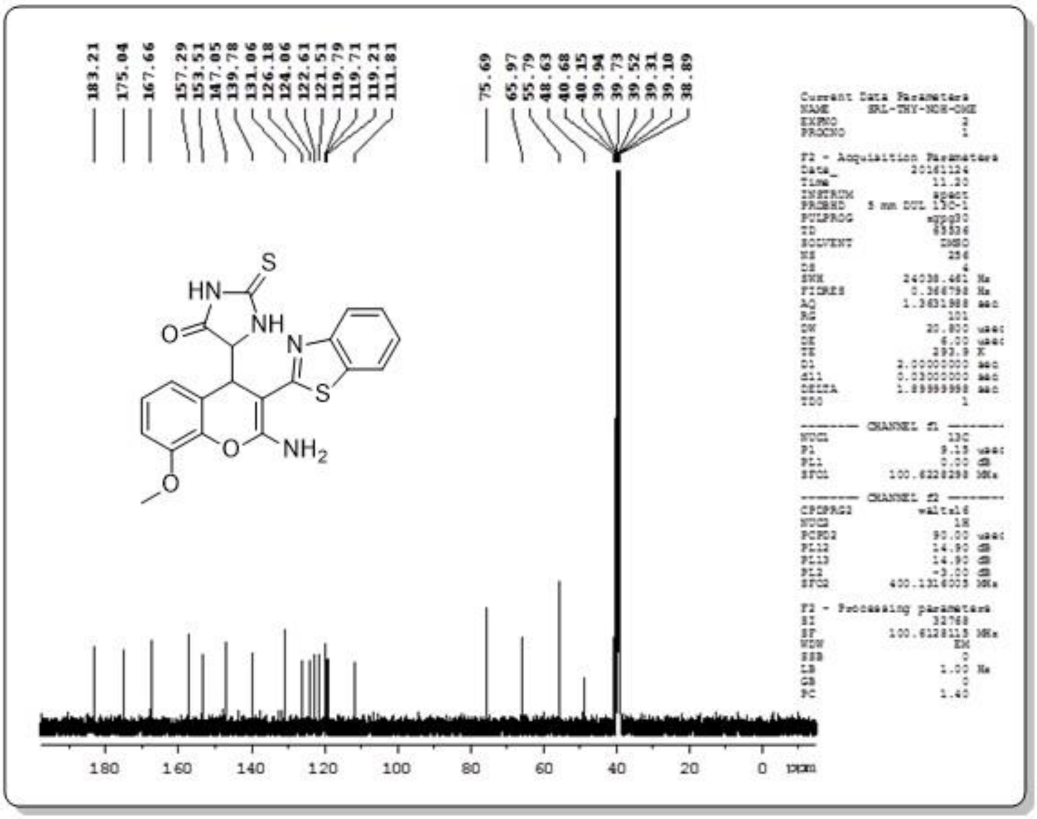

Fig 6: ^{1}H, ^{13}C spectrum of 5-(2-amino-3-(benzo[d]thiazol-2-yl)-8-methoxy-4H-chromen-4-yl)-2-thioxoimidazolidin-4-one **4b**

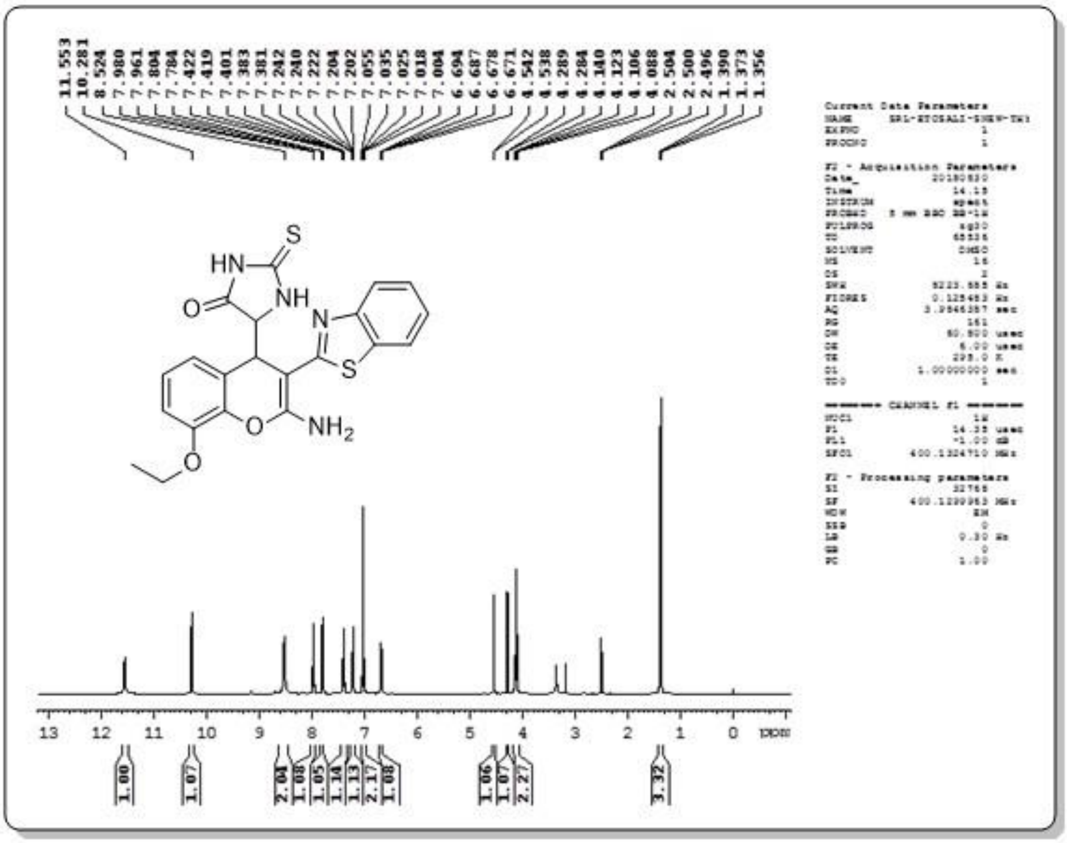

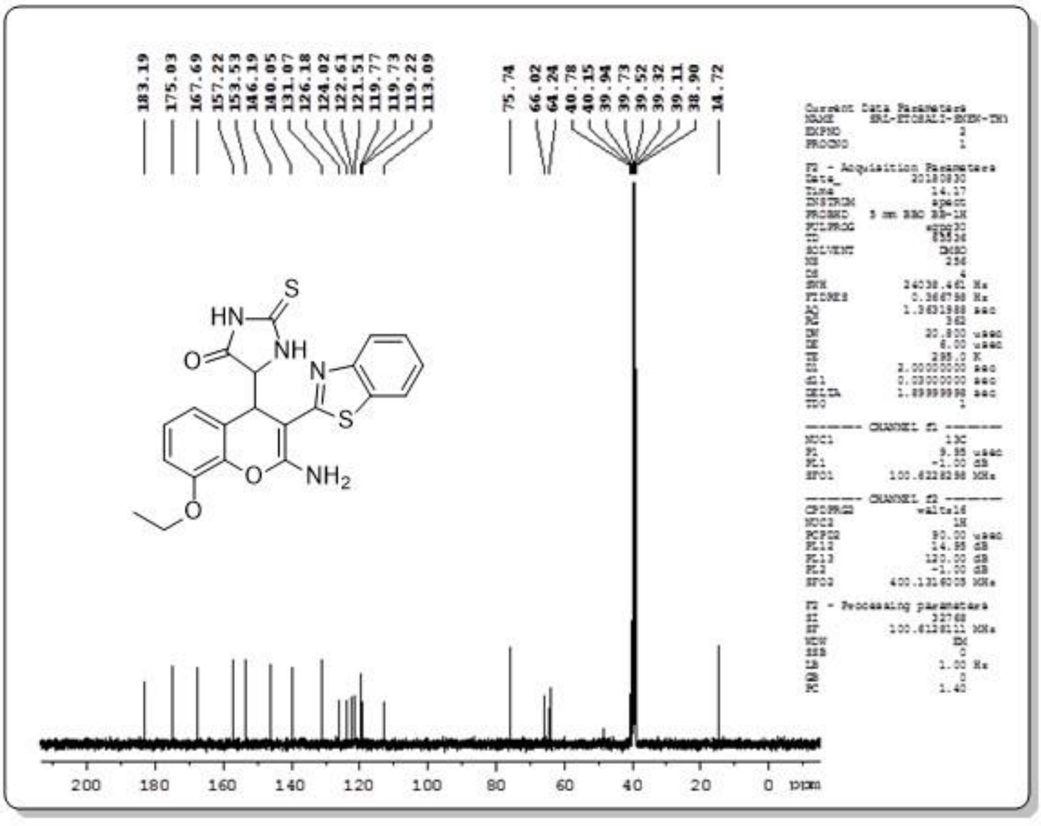

Fig 7: ^{1}H, ^{13}C spectrum of 5-(2-amino-3-(benzo[d]thiazol-2-yl)-8-ethoxy-4H-chromen-4-yl)-2-thioxoimidazolidin-4-one **4c**

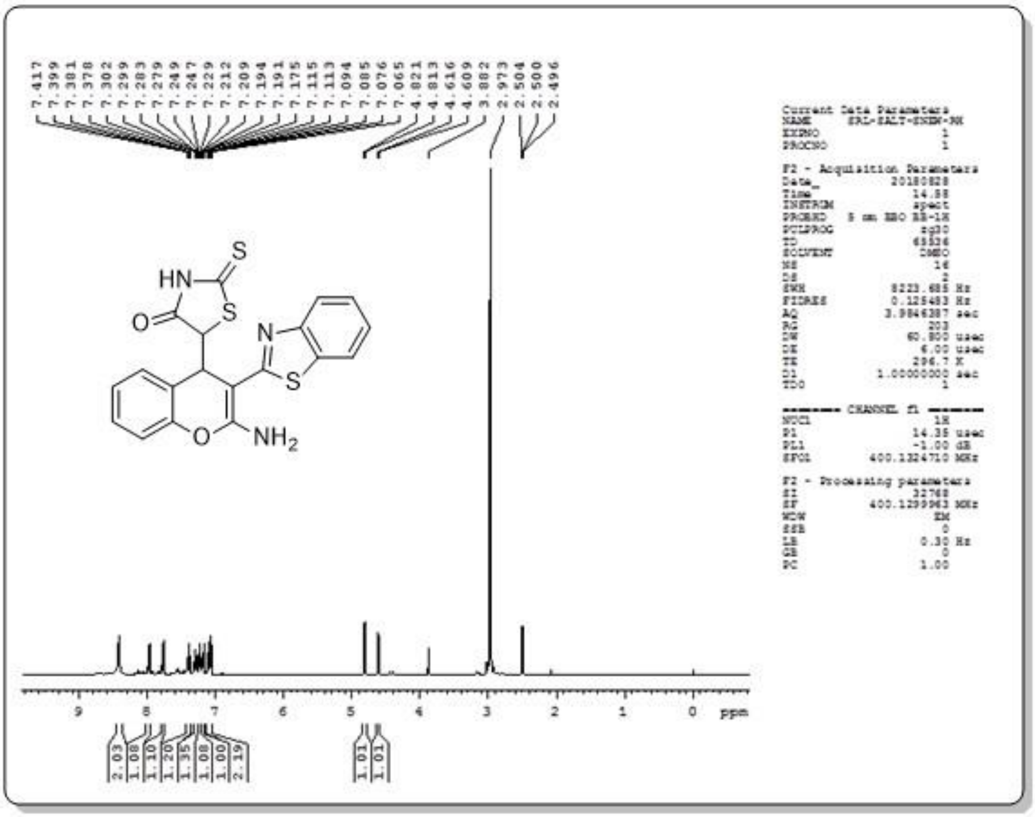

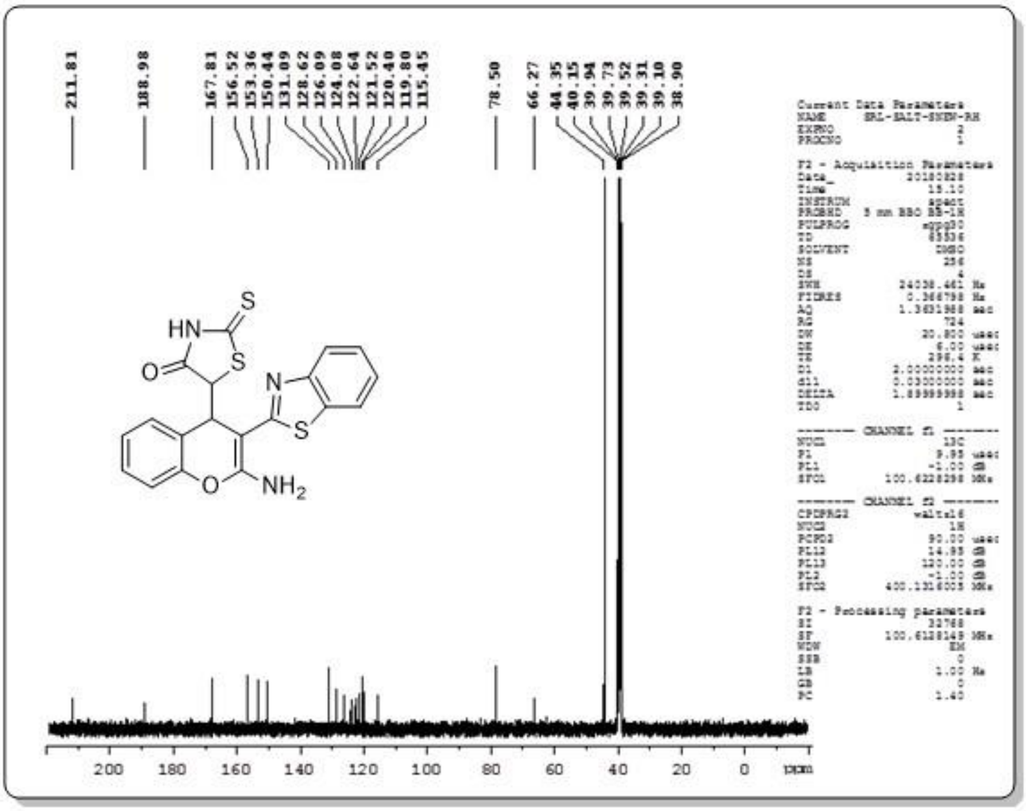

Fig 8: ^{1}H, ^{13}C spectrum of 5-(2-amino-3-(benzo[d]thiazol-2-yl)-4H-chromen-4-yl)-2-thioxothiazolidin-4-one **4d**

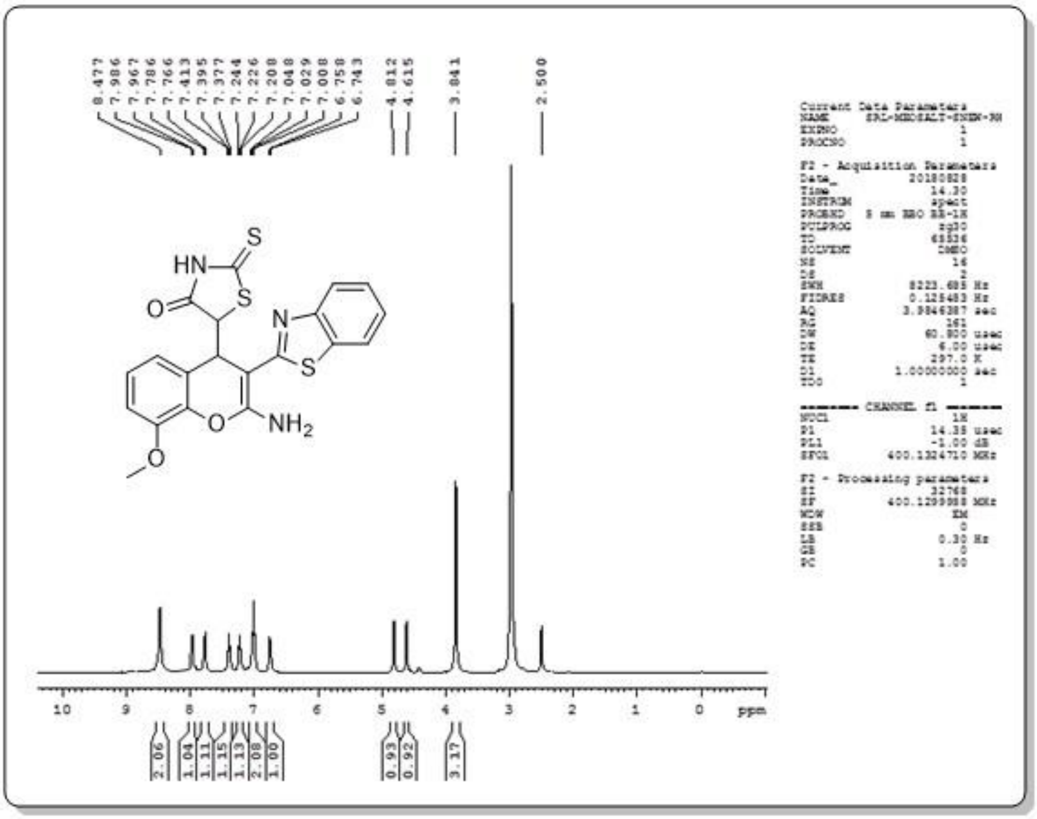

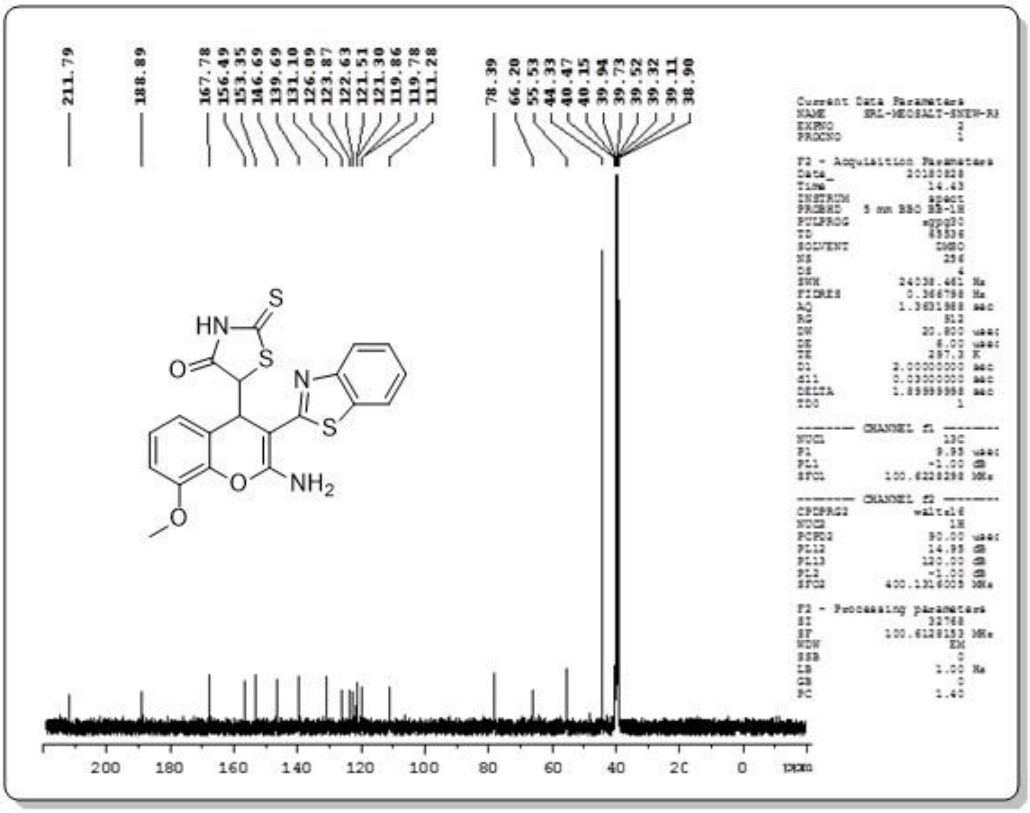

Fig 9: ^{1}H, ^{13}C spectrum of 5-(2-amino-3-(benzo[d]thiazol-2-yl)-8-methoxy-4H-chromen-4-yl)-2-thioxothiazolidin-4-one **4e**

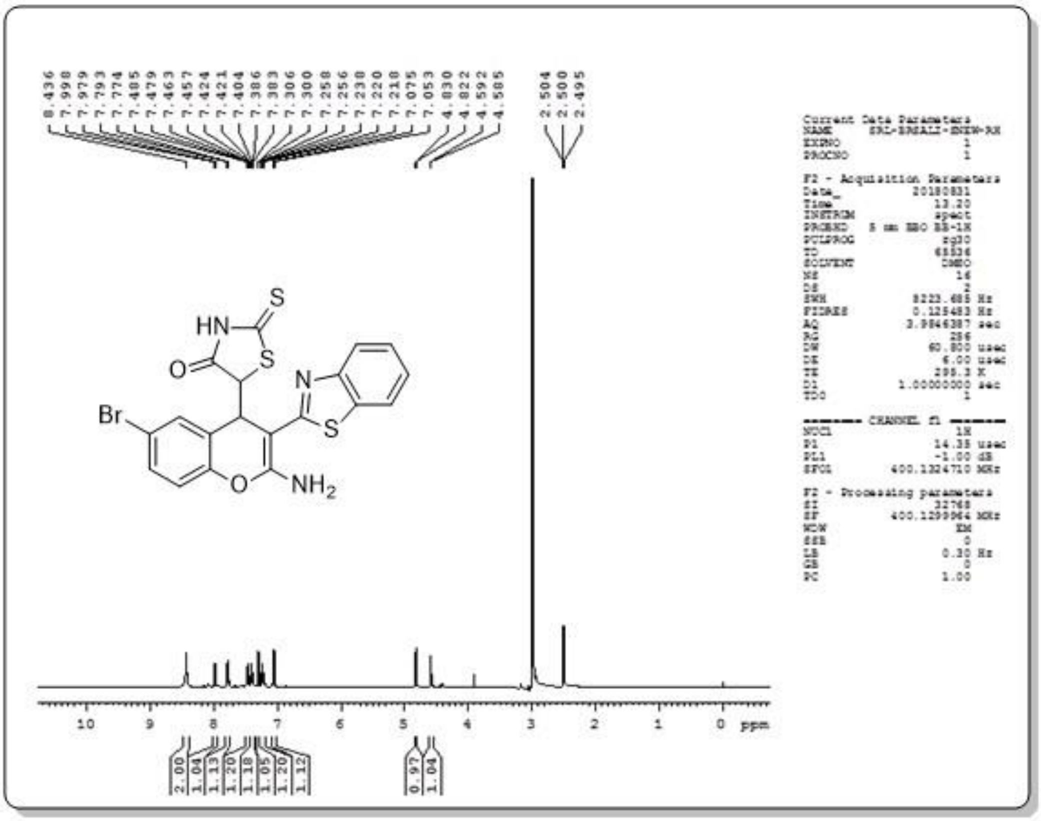

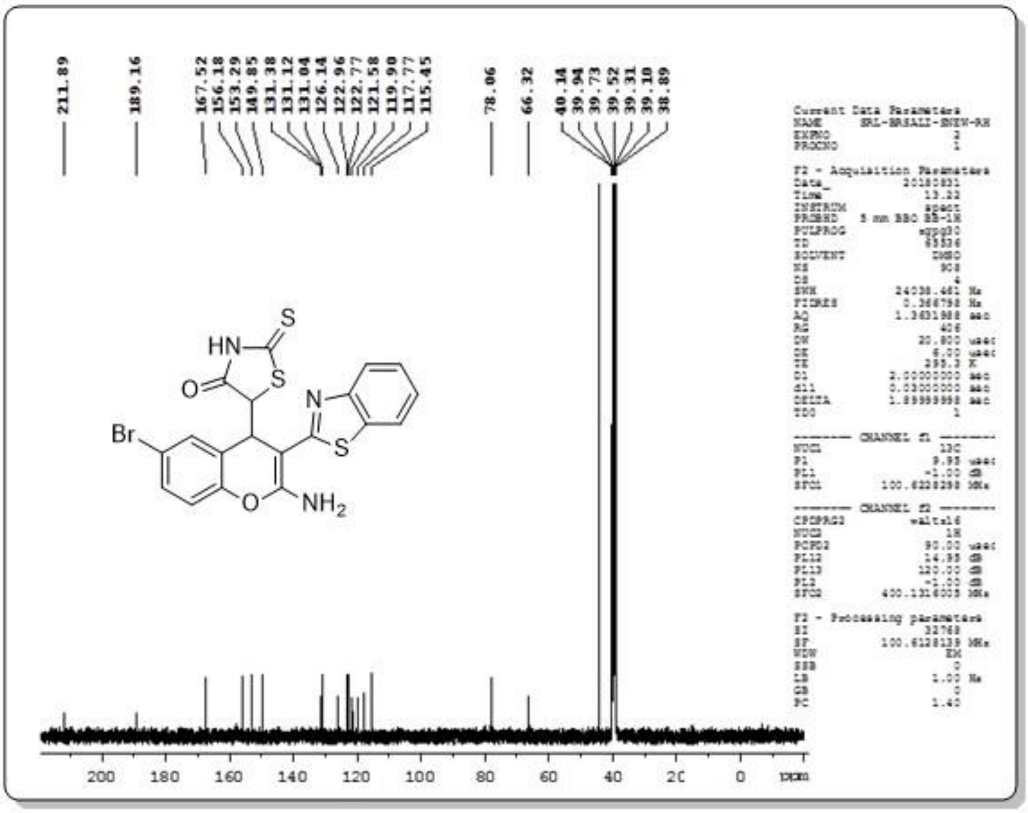

Fig 10: ^{1}H, ^{13}C spectrum of 5-(2-amino-3-(benzo[d]thiazol-2-yl)-6-bromo-4H-chromen-4-yl)-2-thioxothiazolidin-4-one **4f**

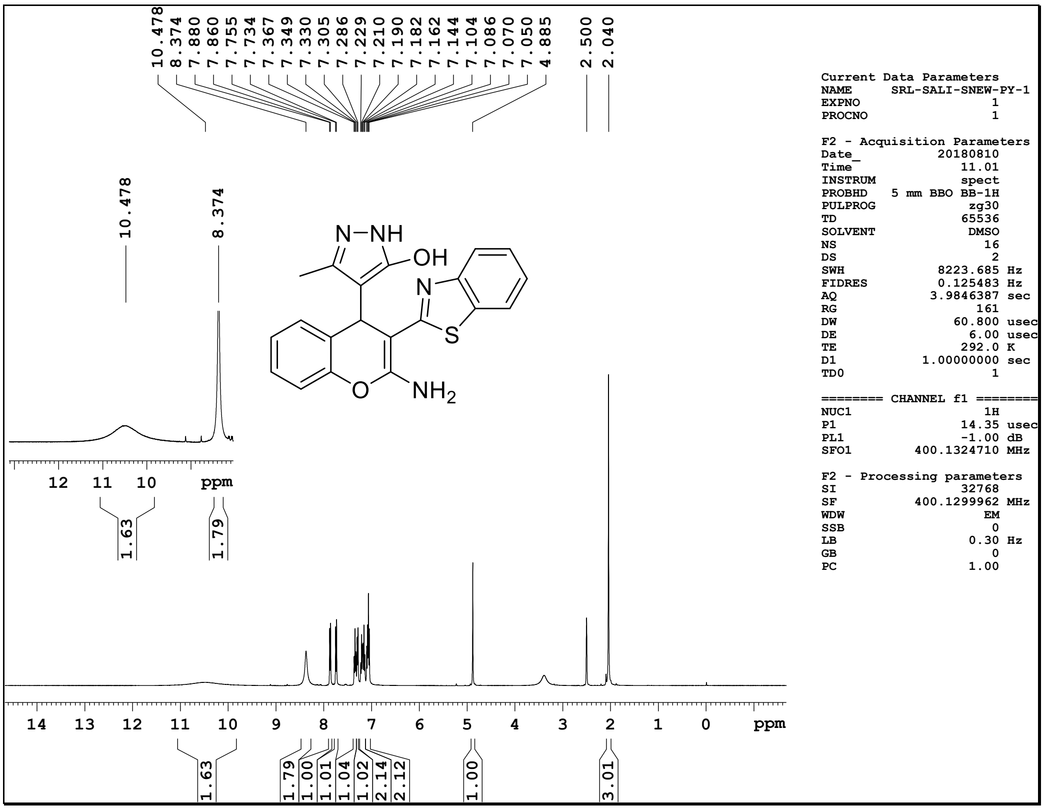

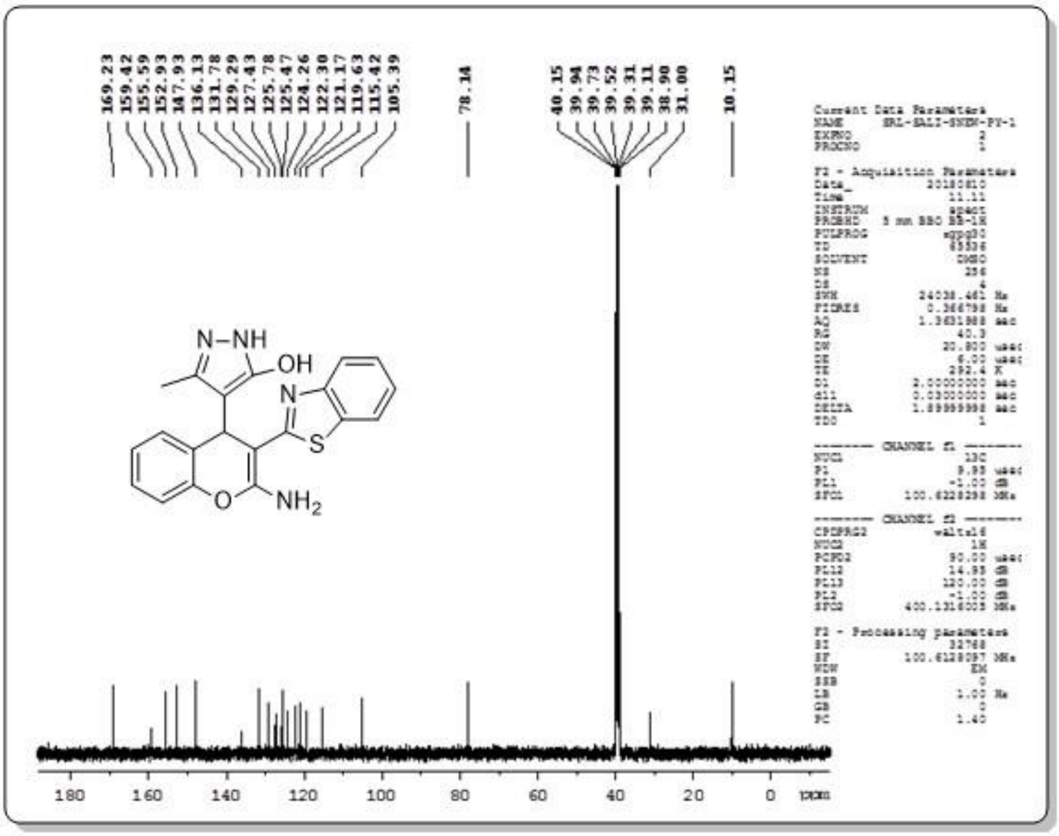

Fig 11. ^{1}H, ^{13}C spectrum of 4-(2-amino-3-(benzo[d]thiazol-2-yl)-4H-chromen-4-yl)-3-methyl-1H-pyrazol-5-ol **4g**

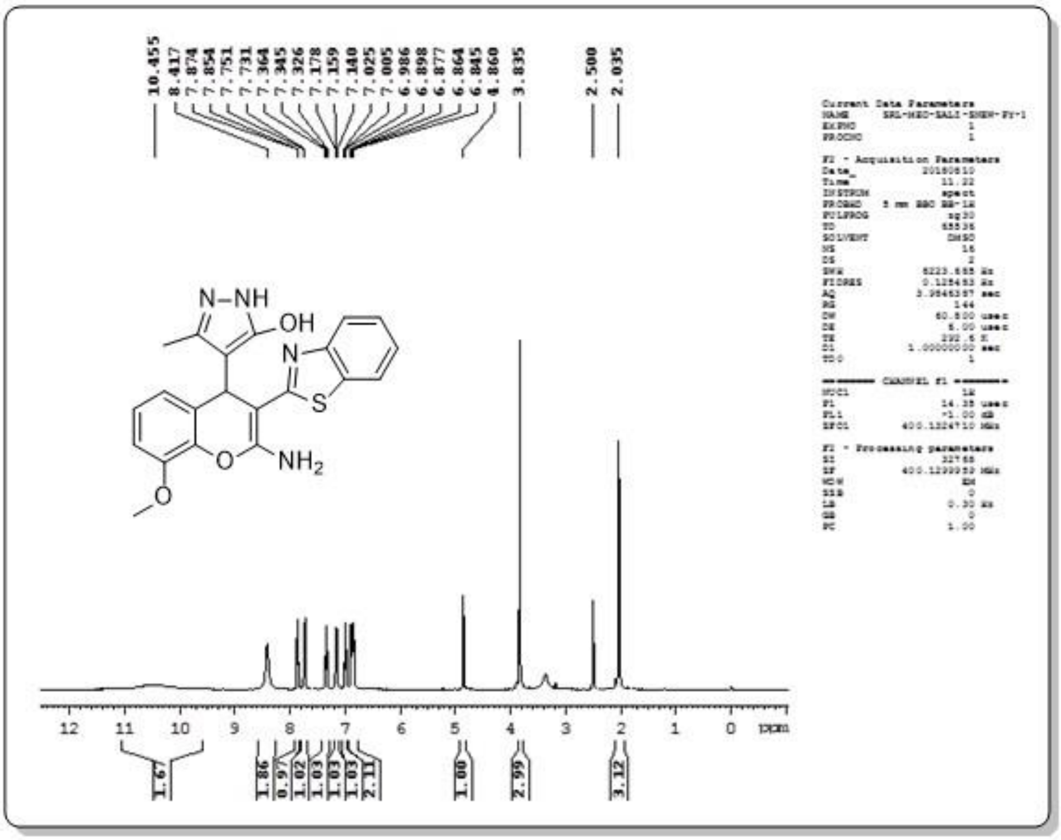

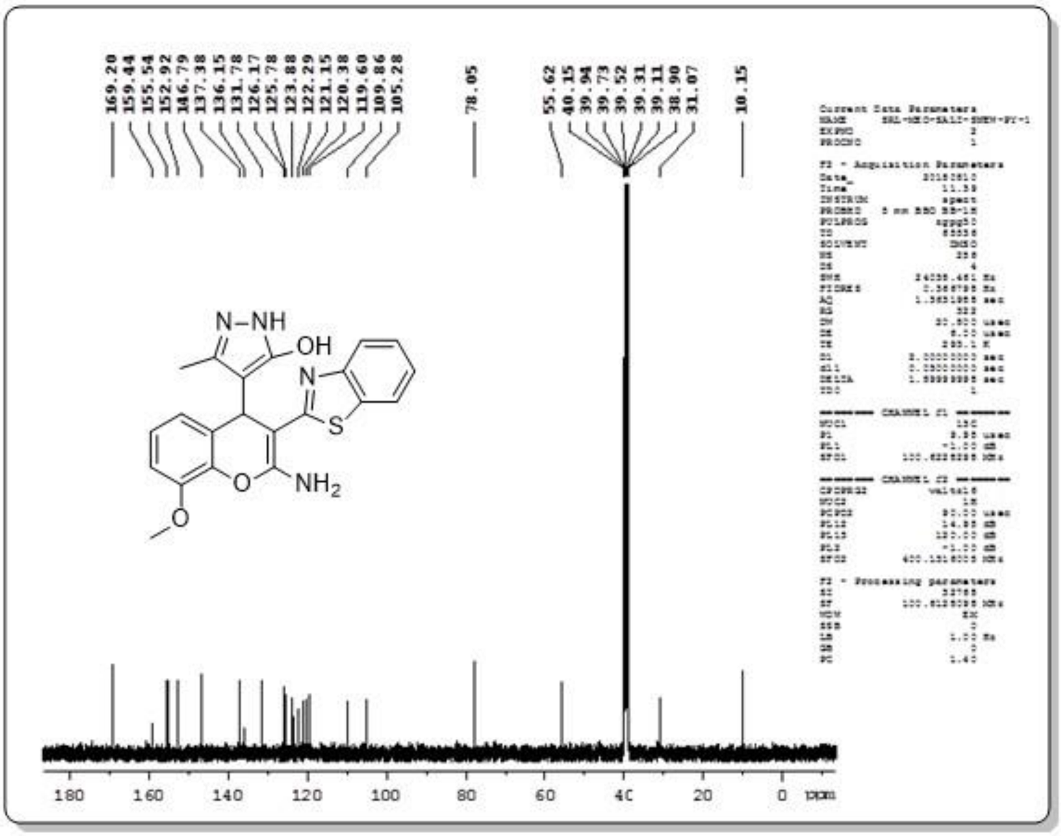

Fig 12: ^{1}H, ^{13}C spectrum of 4-(2-amino-3-(benzo[d]thiazol-2-yl)-8-methoxy-4H-chromen-4-yl)-3-methyl-1H-pyrazol-5-ol **4h**

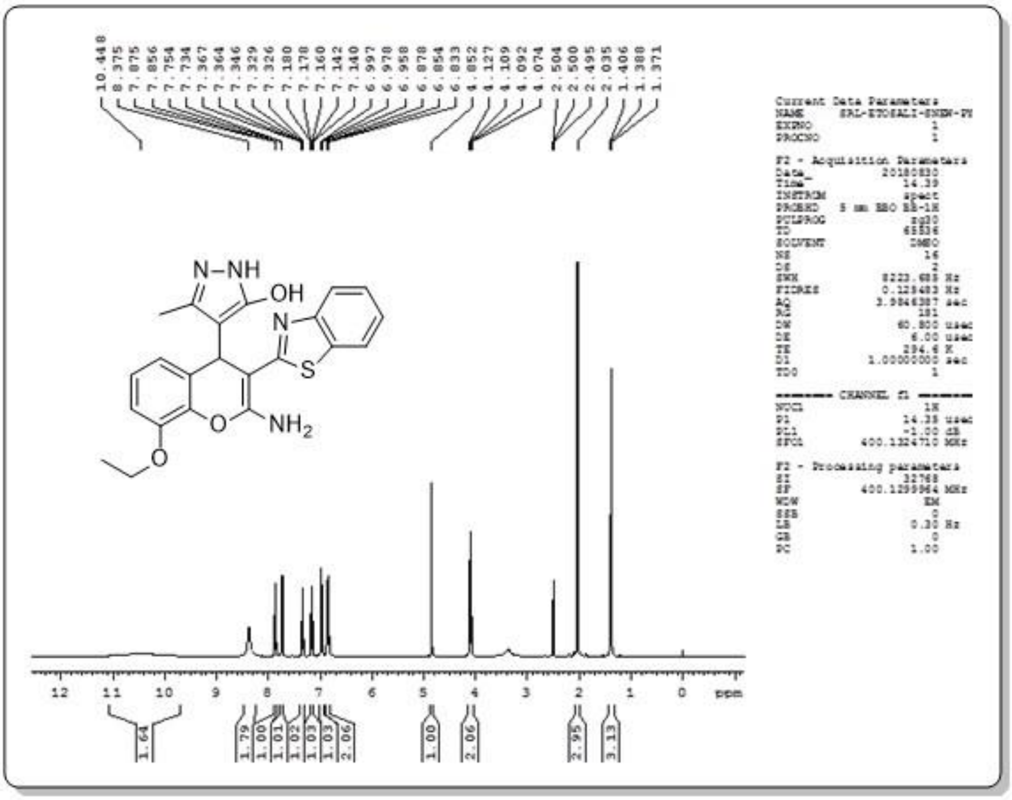

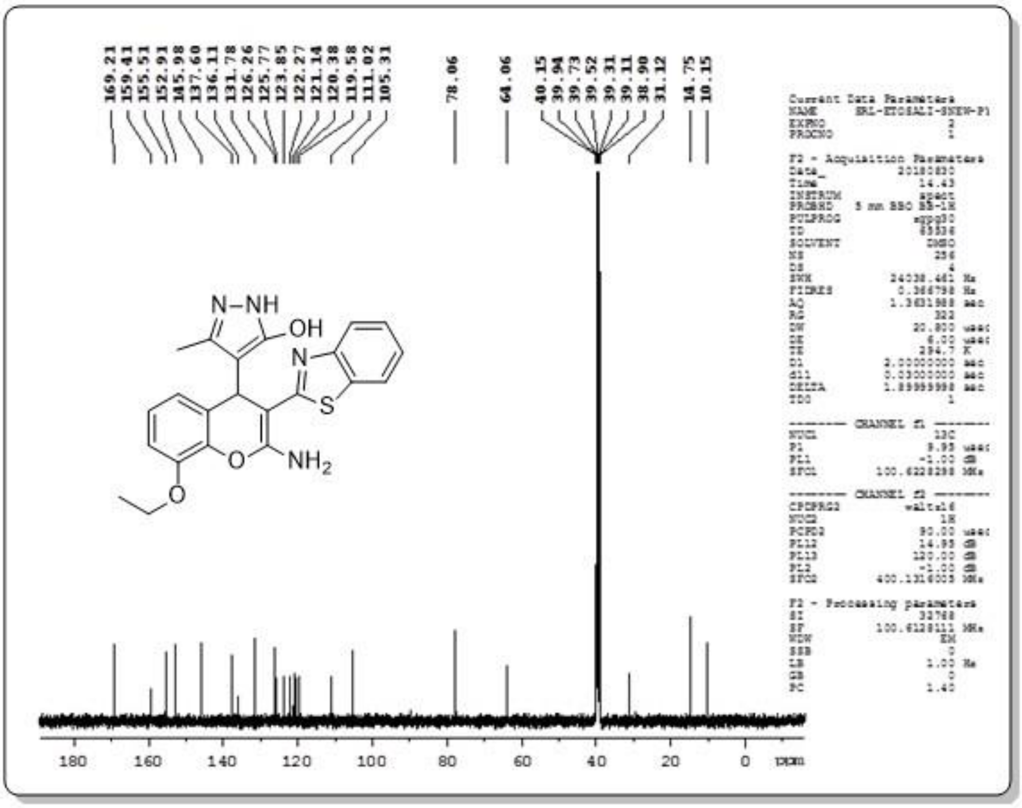

Fig 13: ^{1}H, ^{13}C spectrum of 4-(2-amino-3-(benzo[d]thiazol-2-yl)-8-ethoxy-4H-chromen-4-yl)-3-methyl-1H-pyrazol-5-ol **4i**

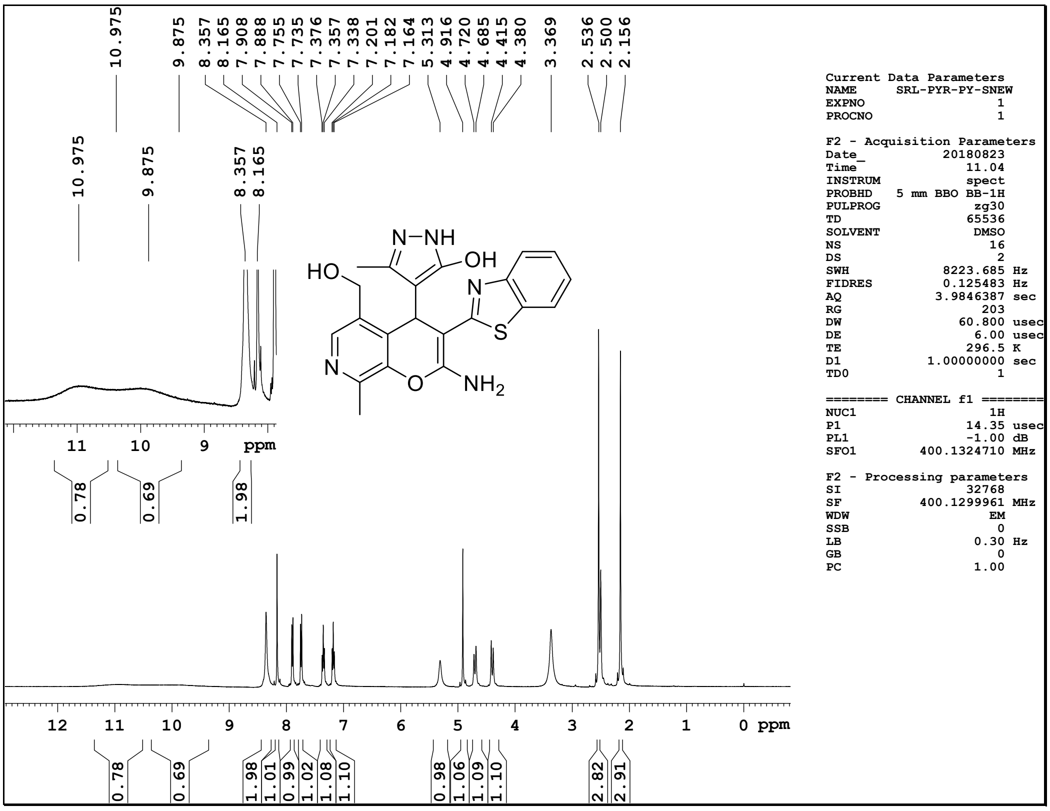

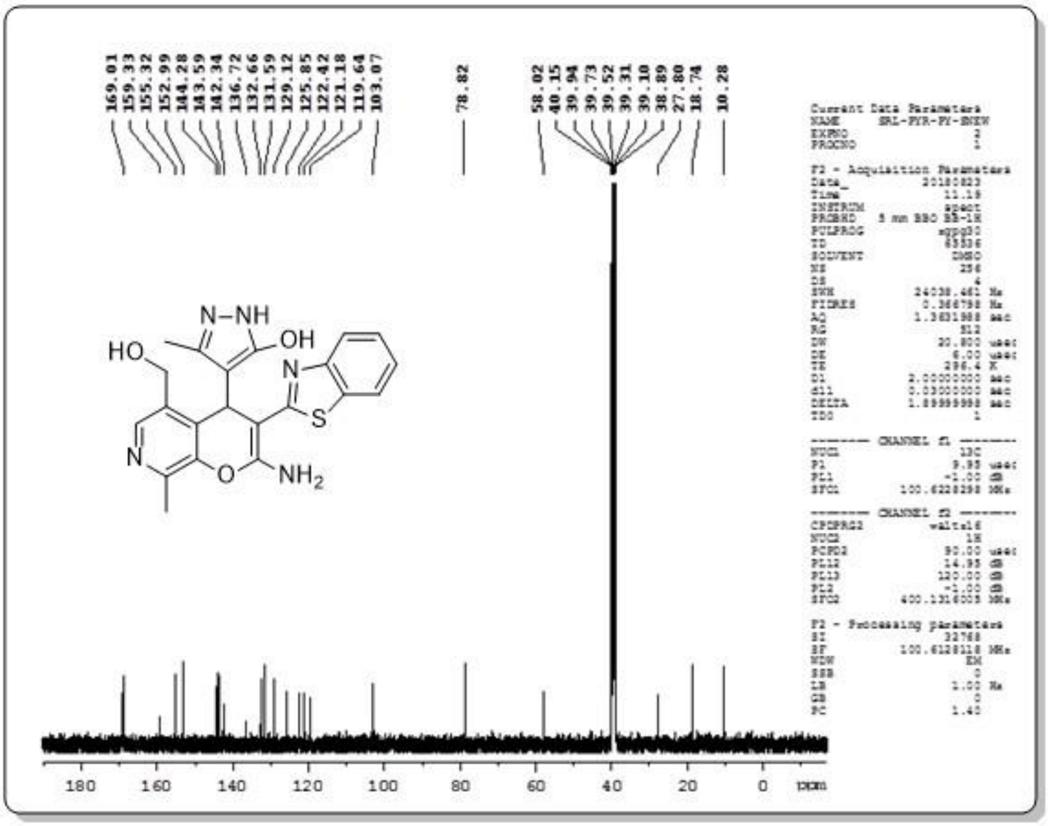

Fig 14: ^{1}H, ^{13}C spectrum of 4-(2-amino-3-(benzo[d]thiazol-2-yl)-5-(hydroxymethyl)-8-methyl-4H-pyrano[2,3-c]pyridin-4-yl)-3-methyl-1H-pyrazol-5-ol **4j**

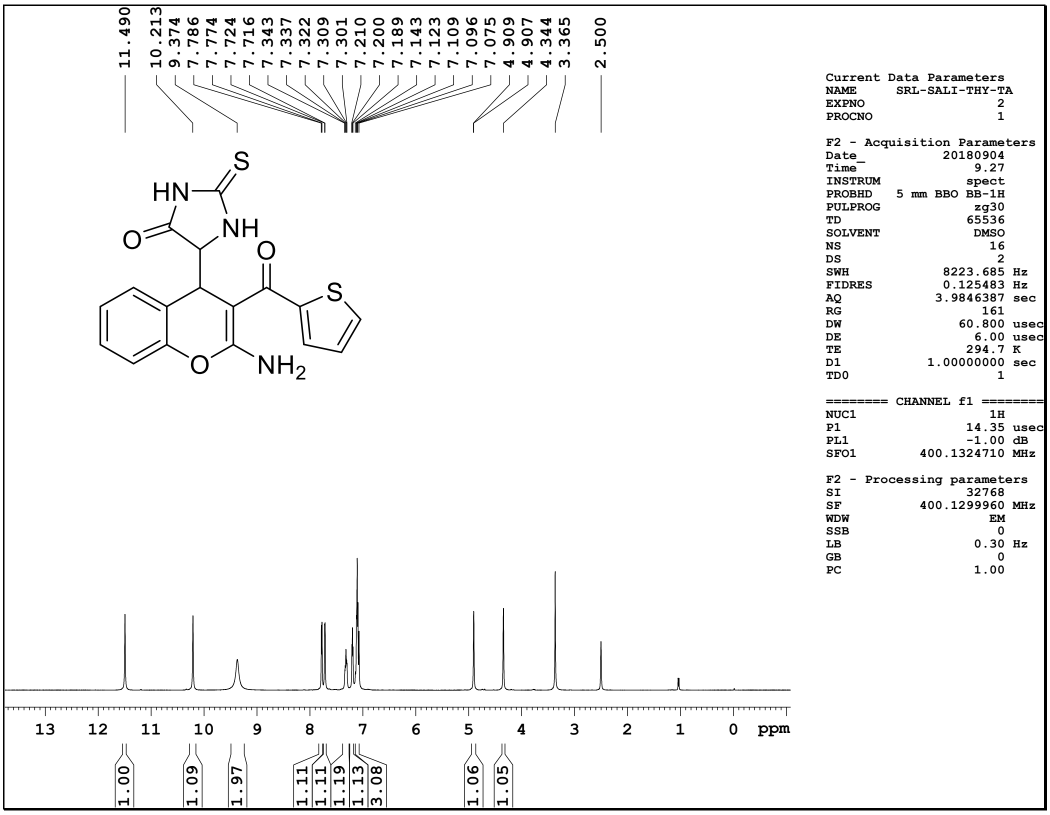

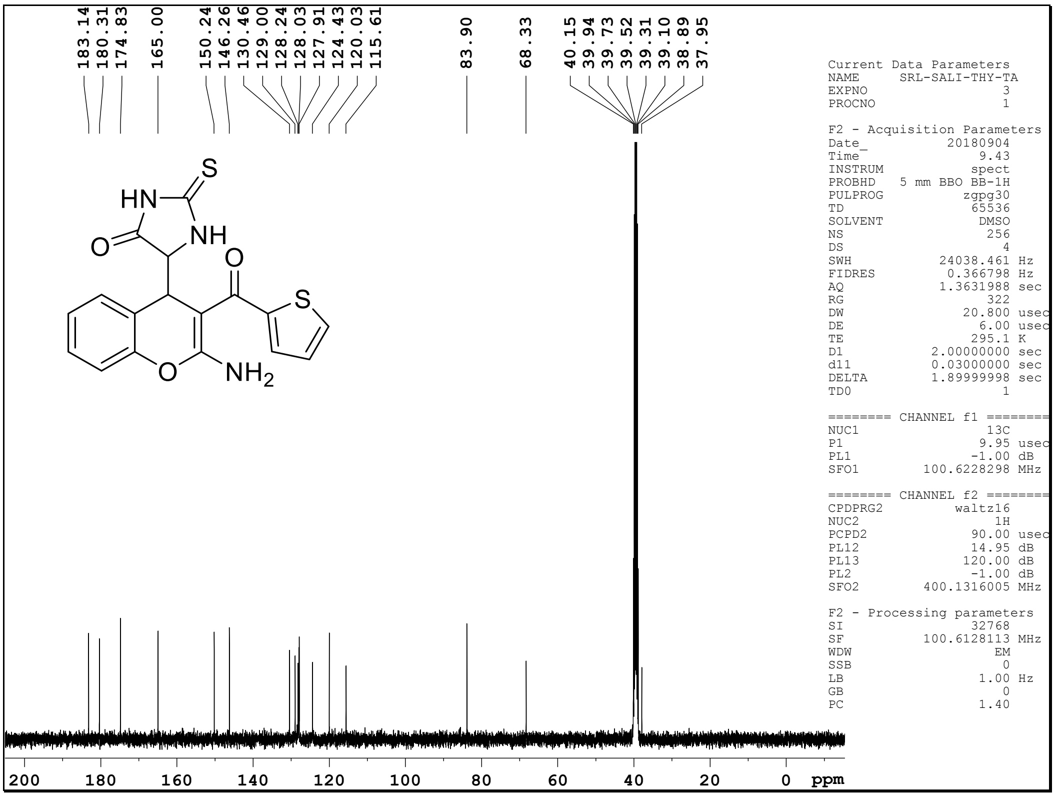

Fig 15: ^{1}H, ^{13}C spectrum of 5-(2-amino-3-(thiophene-2-carbonyl)-4H-chromen-4-yl)-2-thioxoimidazolidin-4-one **7a**

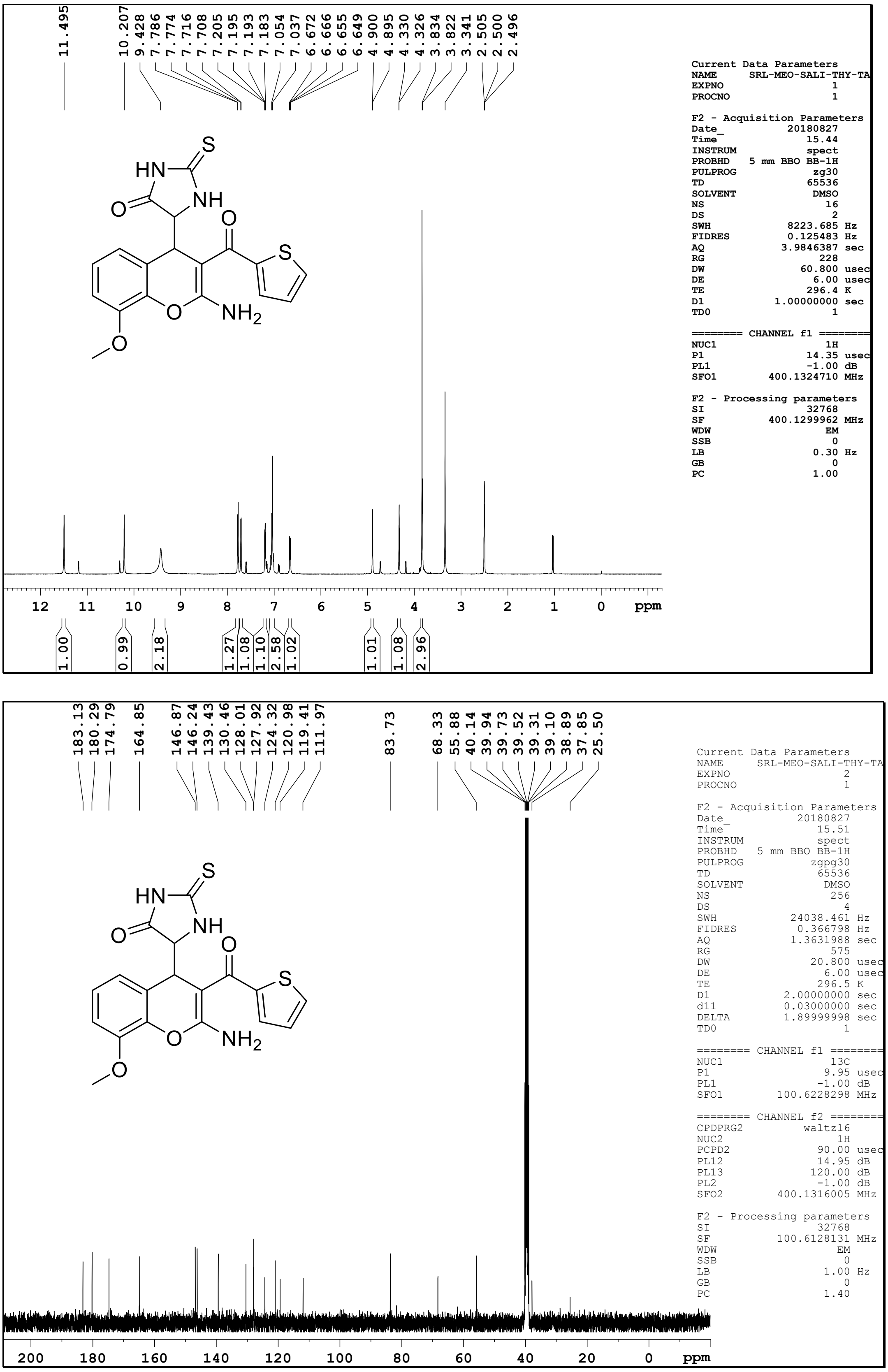

Fig 16: ^{1}H, ^{13}C spectrum of 5-(2-amino-8-methoxy-3-(thiophene-2-carbonyl)-4H-chromen-4-yl)-2-thioxoimidazolidin-4-one **7b**

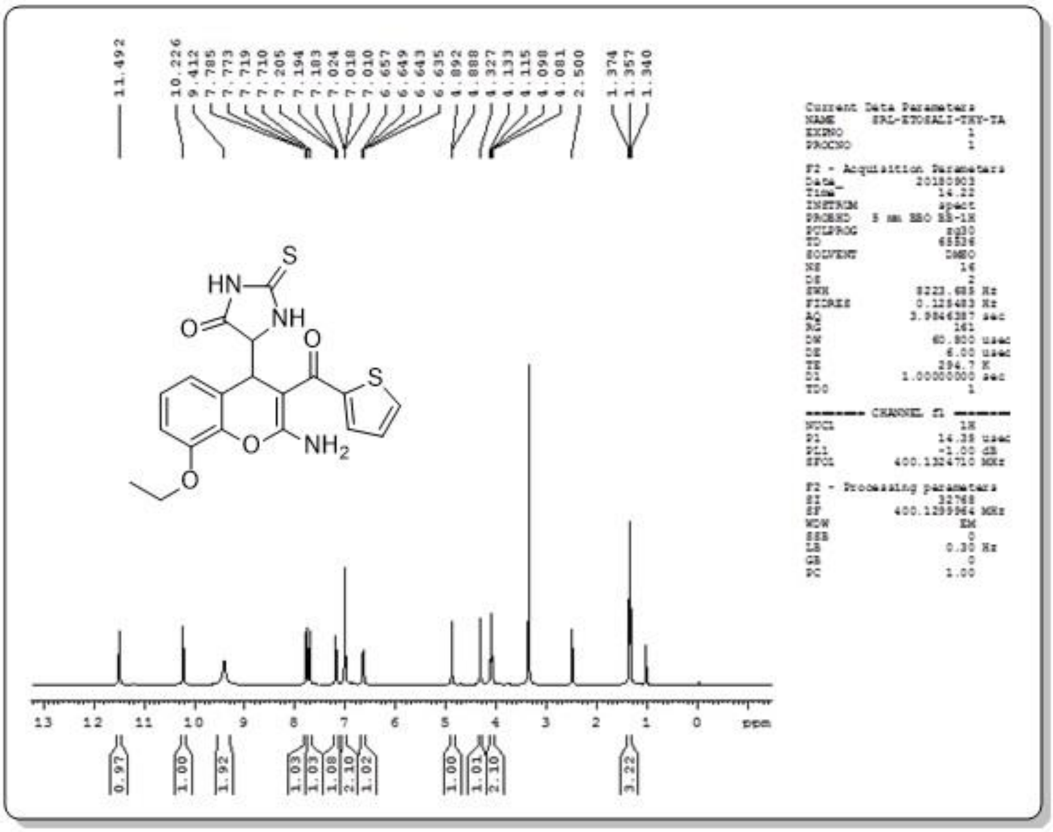

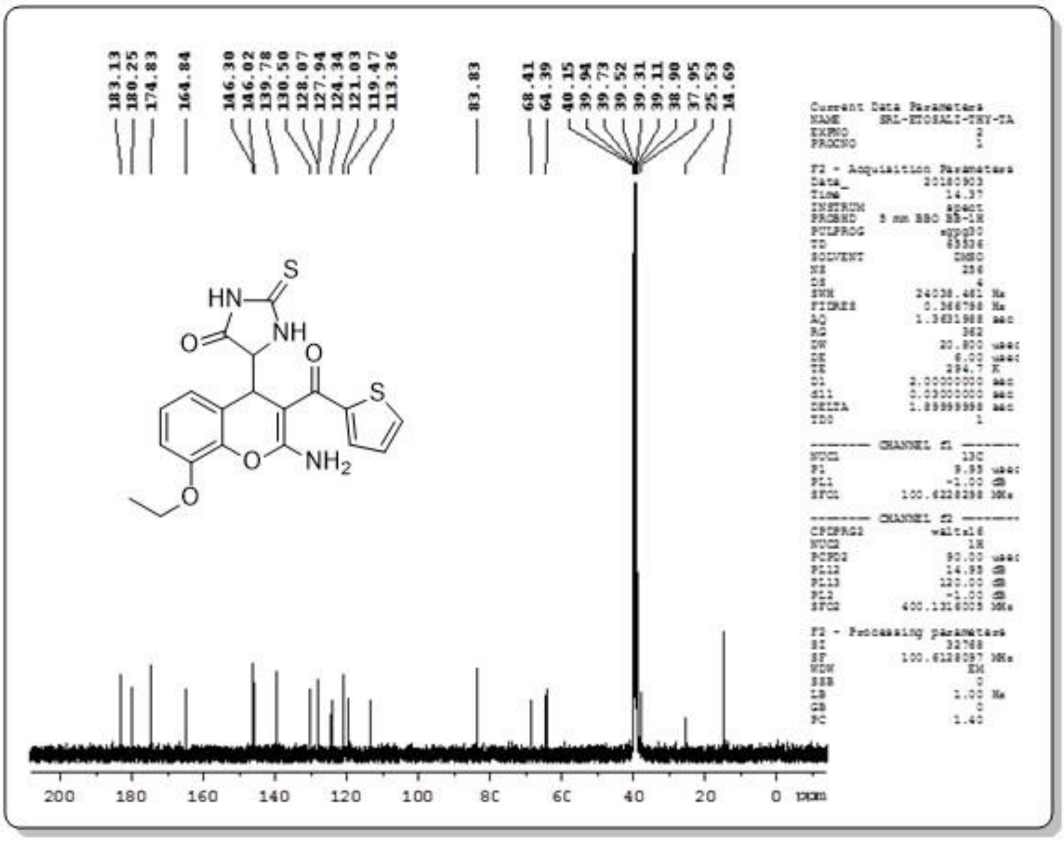

Fig 17: ^{1}H, ^{13}C spectrum of 5-(2-amino-8-ethoxy-3-(thiophene-2-carbonyl)-4H-chromen-4-yl)-2-thioxoimidazolidin-4-one **7c**

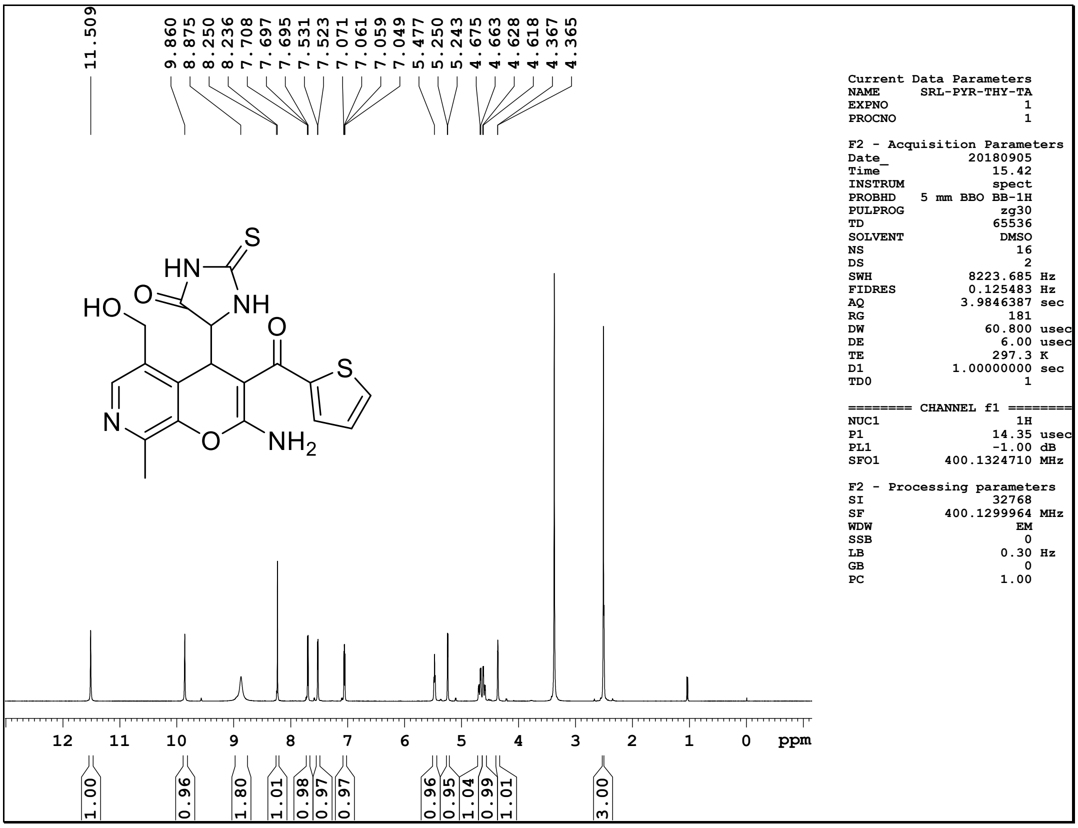

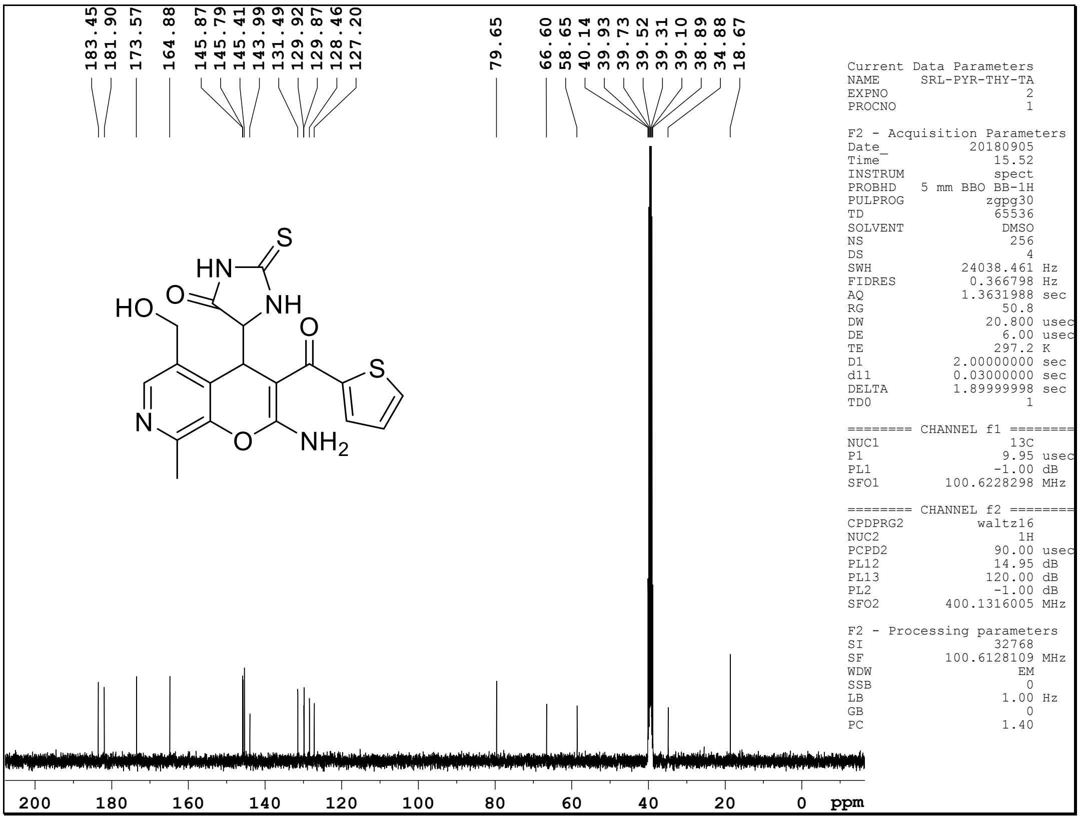

Fig 18: ^{1}H, ^{13}C spectrum of 5-(2-amino-5-(hydroxymethyl)-8-methyl-3-(thiophene-2-carbonyl)-4H-pyrano[2,3-c]pyridin-4-yl)-2-thioxoimidazolidin-4-one **7d**

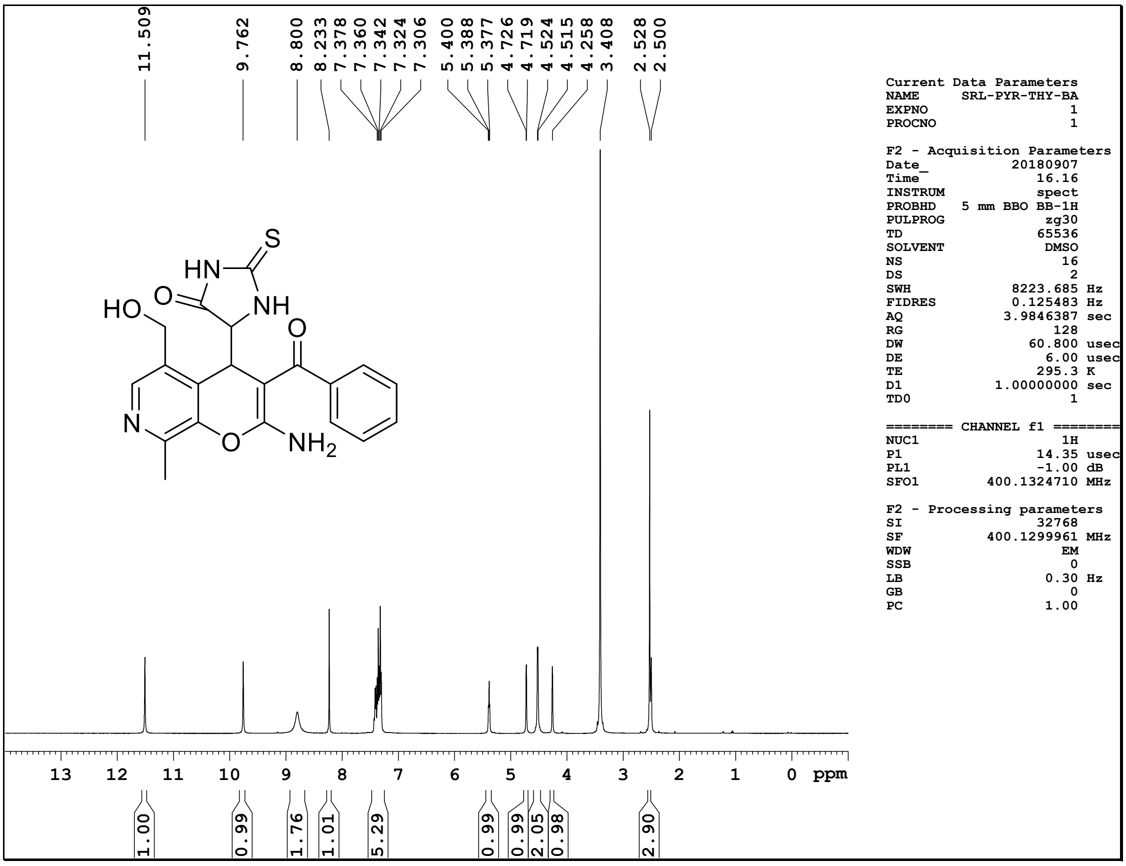

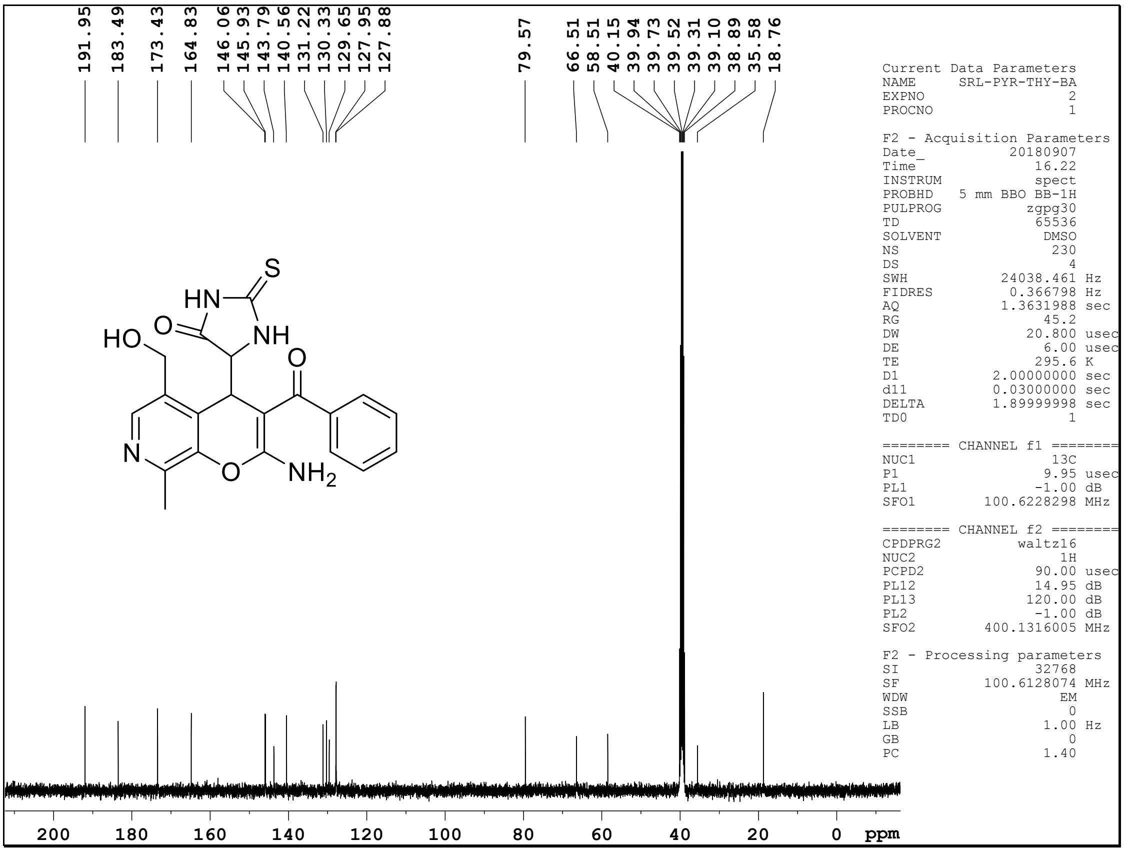

Fig 19: ^{1}H, ^{13}C spectrum of 5-(2-amino-3-benzoyl-5-(hydroxymethyl)-8-methyl-4H-pyrano[2,3-c]pyridin-4-yl)-2-thioxoimidazolidin-4-one **7e**

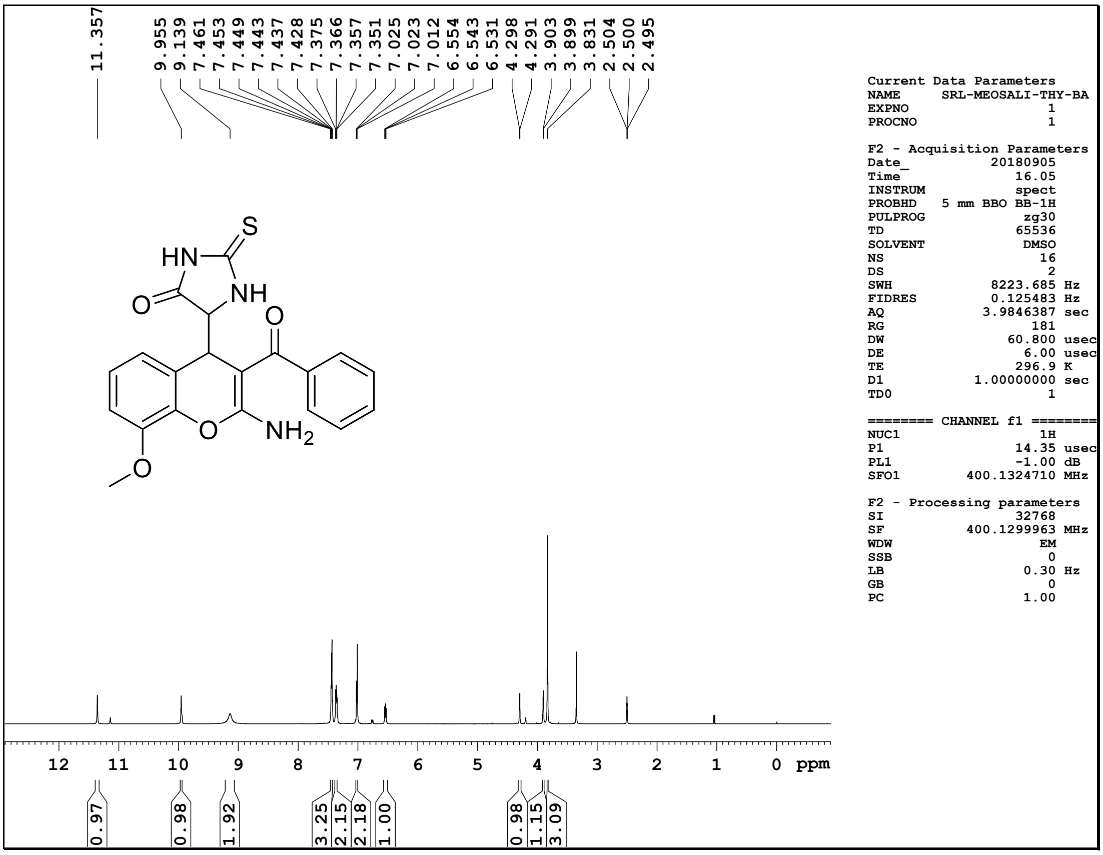

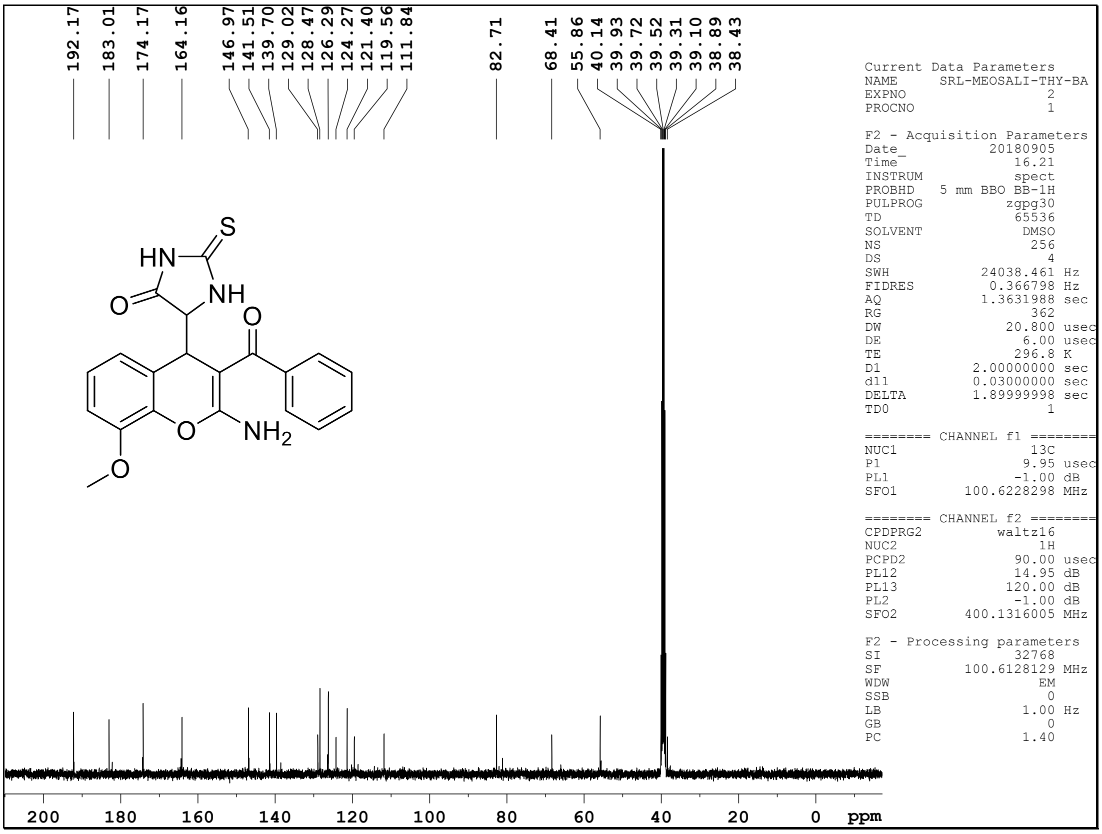

Fig 20: ^{1}H, ^{13}C spectrum of 5-(2-amino-3-benzoyl-8-methoxy-4H-chromen-4-yl)-2-thioxoimidazolidin-4-one **7f**

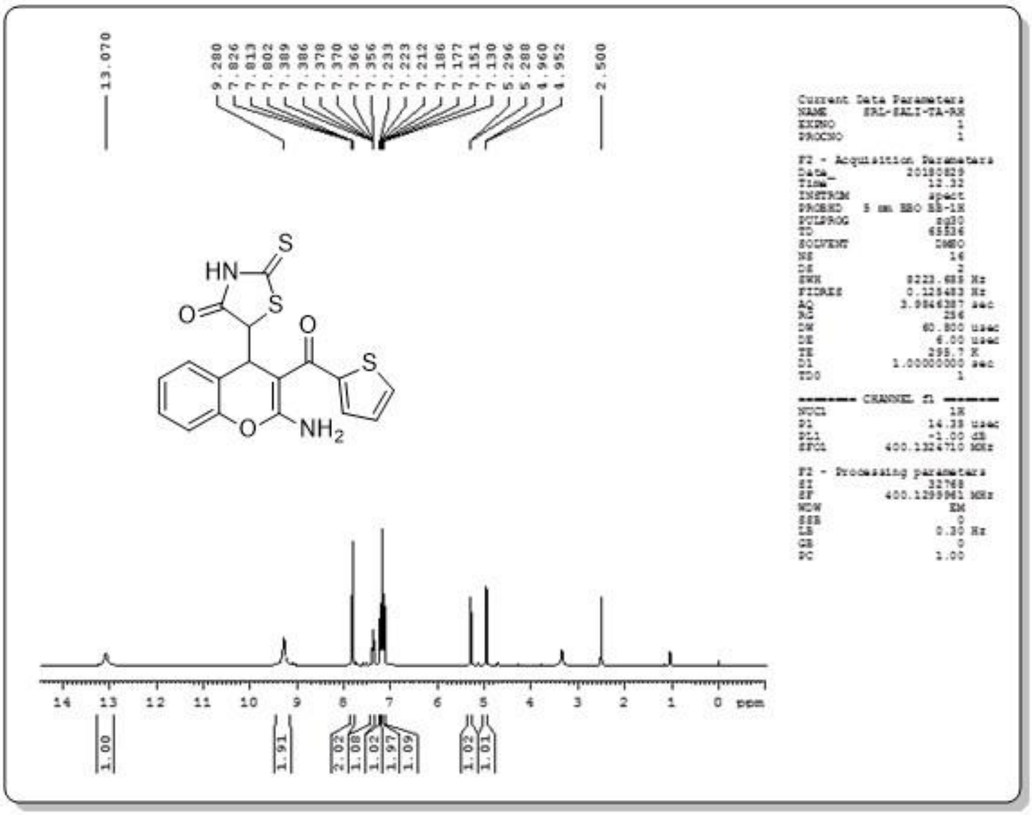

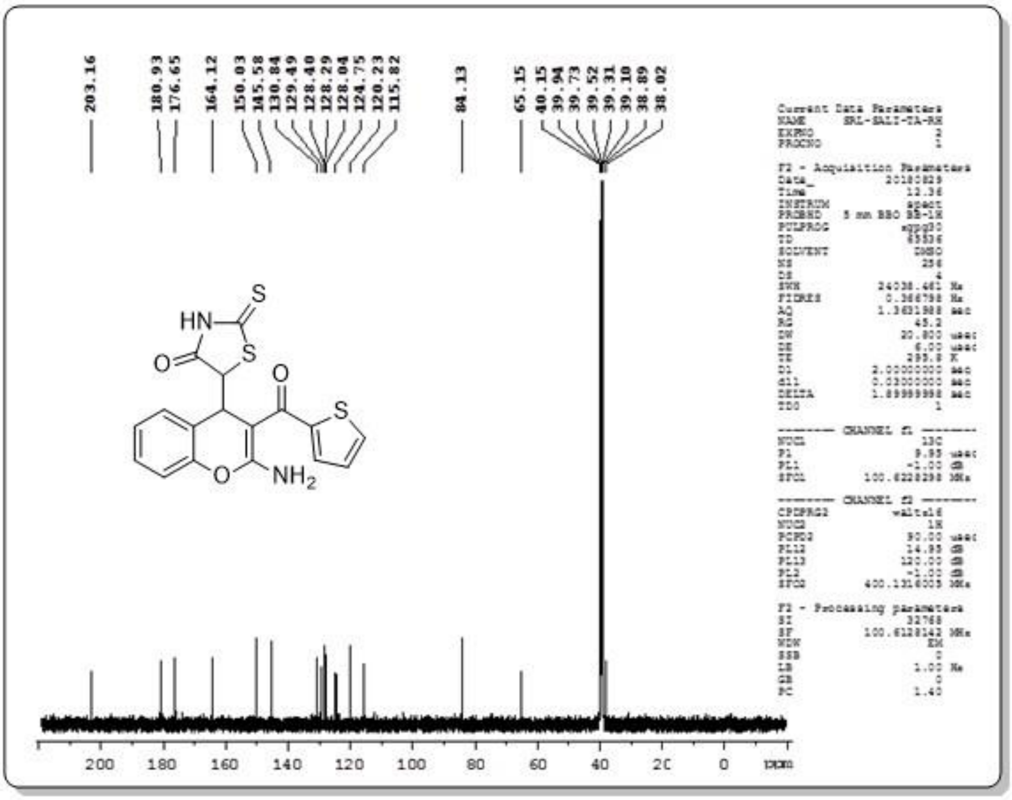

Fig 21: ^{1}H, ^{13}C spectrum of 5-(2-amino-3-(thiophene-2-carbonyl)-4H-chromen-4-yl)-2-thioxothiazolidin-4-one **7g**

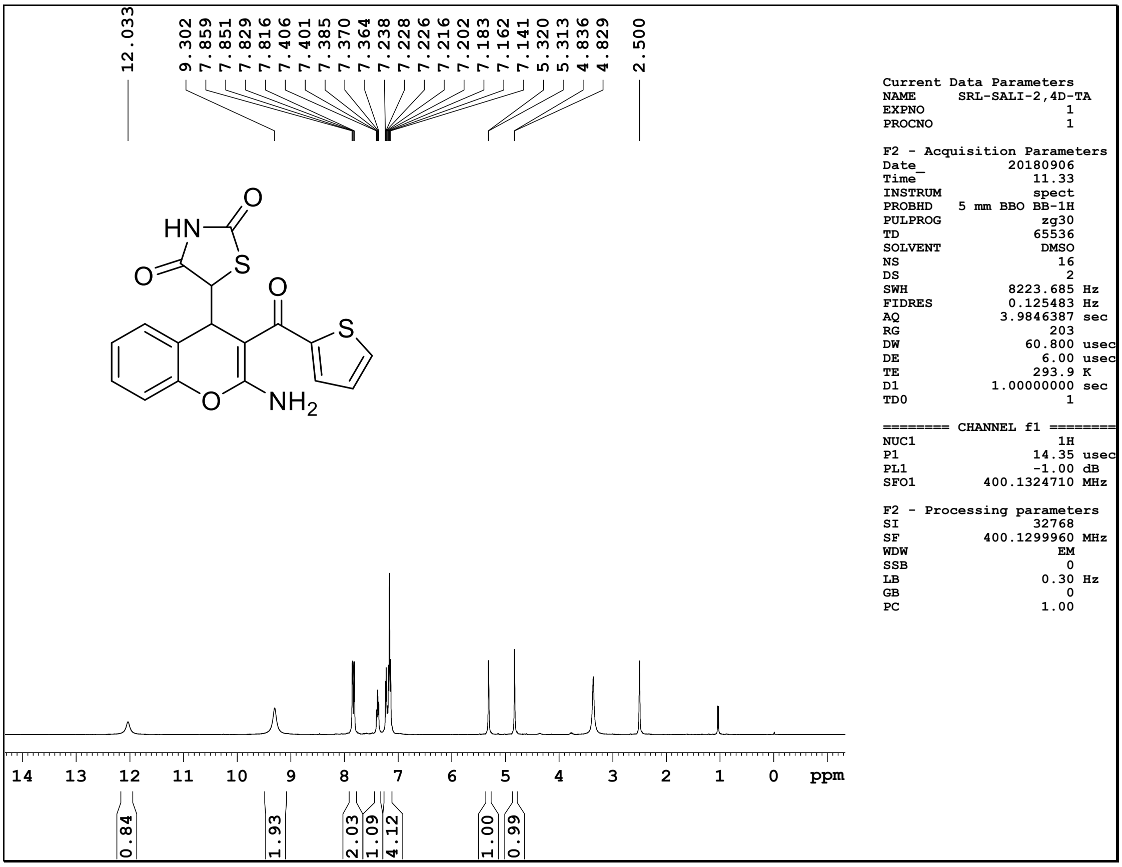

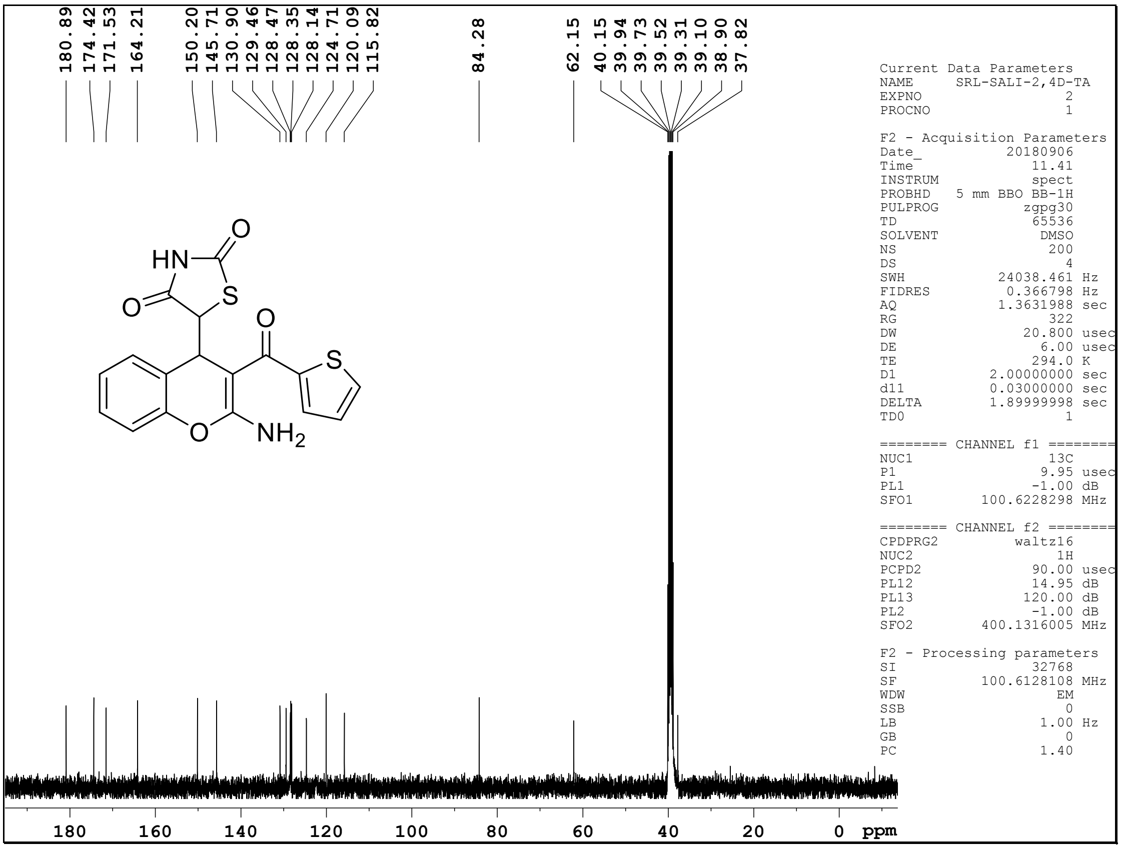

Fig 22: ^{1}H, ^{13}C spectrum of 5-(2-amino-3-(thiophene-2-carbonyl)-4H-chromen-4-yl)thiazolidine-2,4-dione **7h**

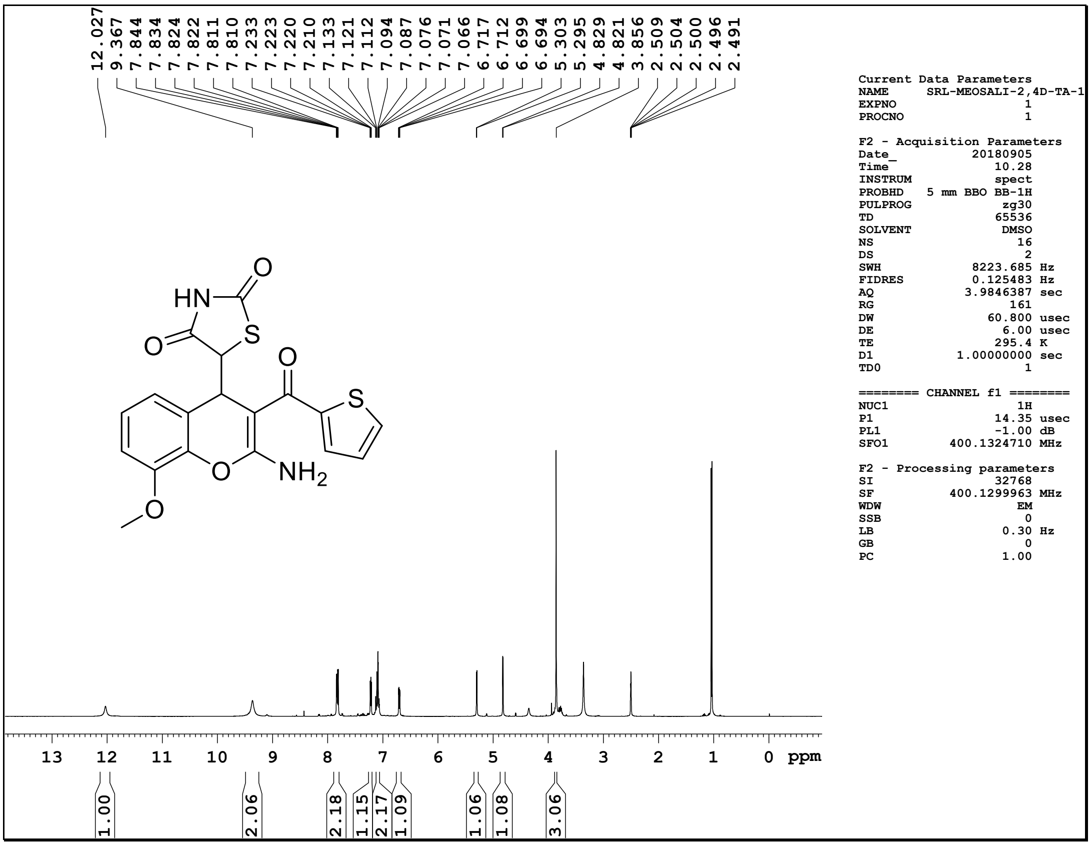

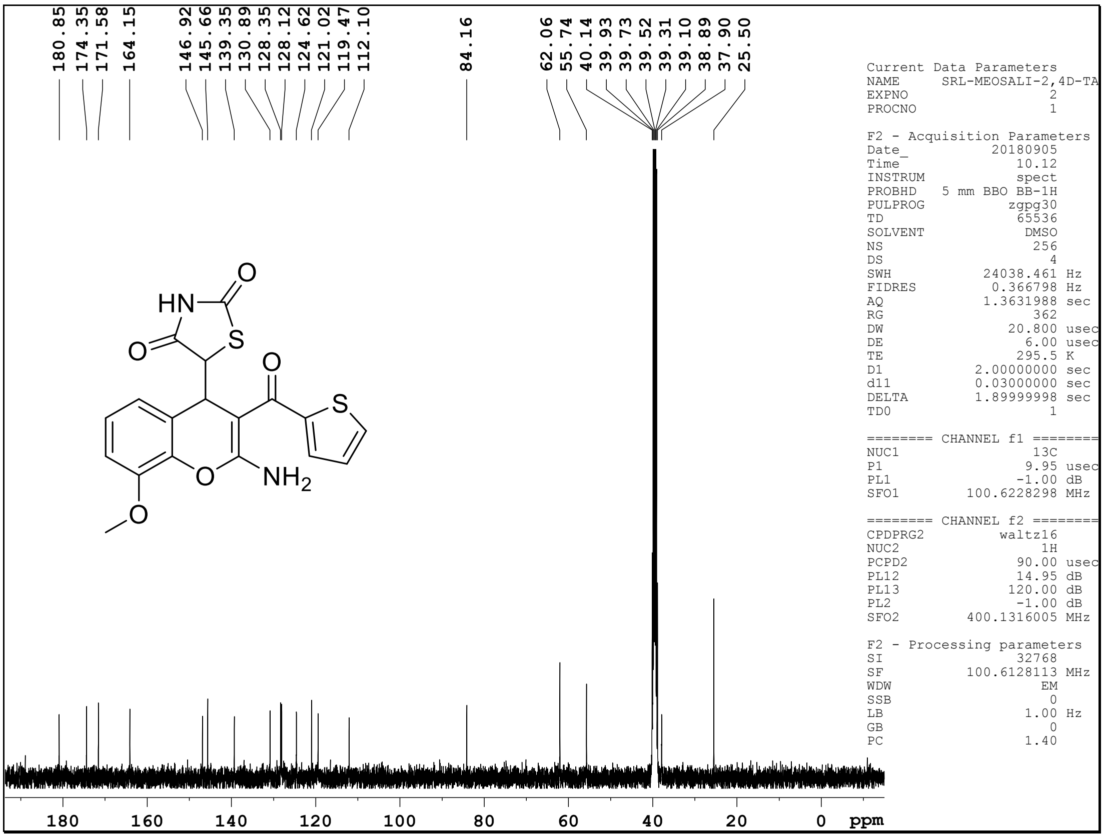

Fig 23: ^{1}H, ^{13}C spectrum of 5-(2-amino-8-methoxy-3-(thiophene-2-carbonyl)-4H-chromen-4-yl)thiazolidine-2,4-dione **7i**

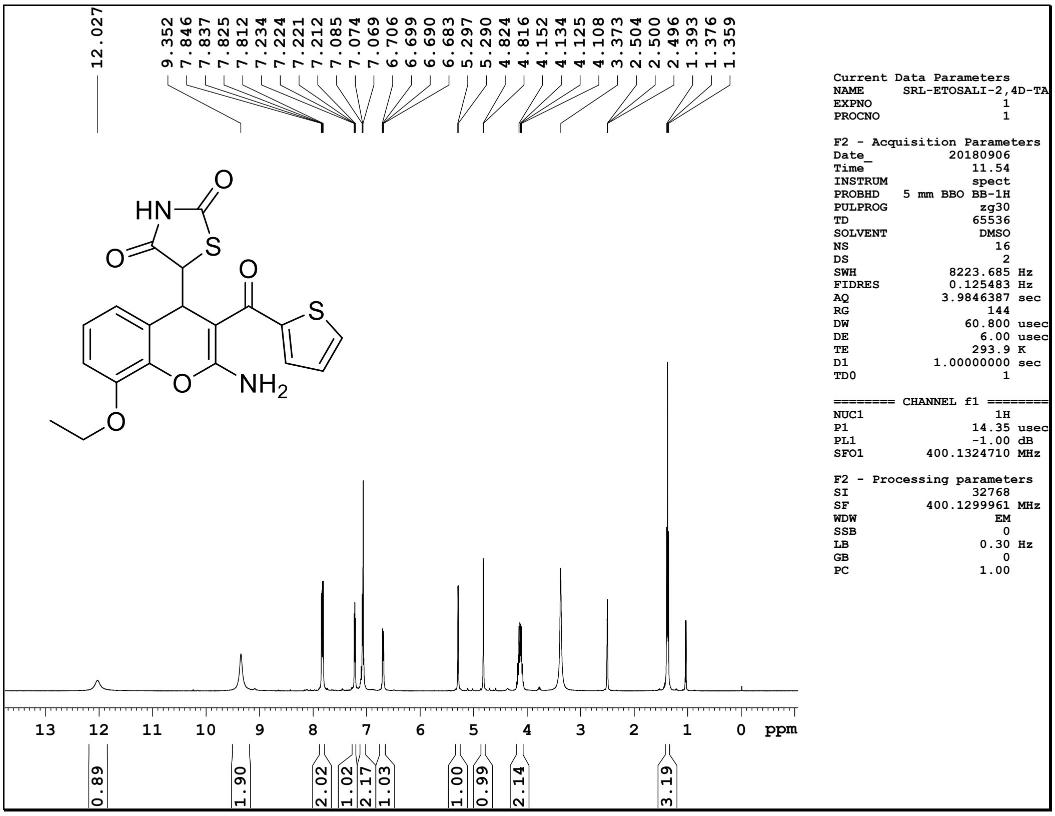

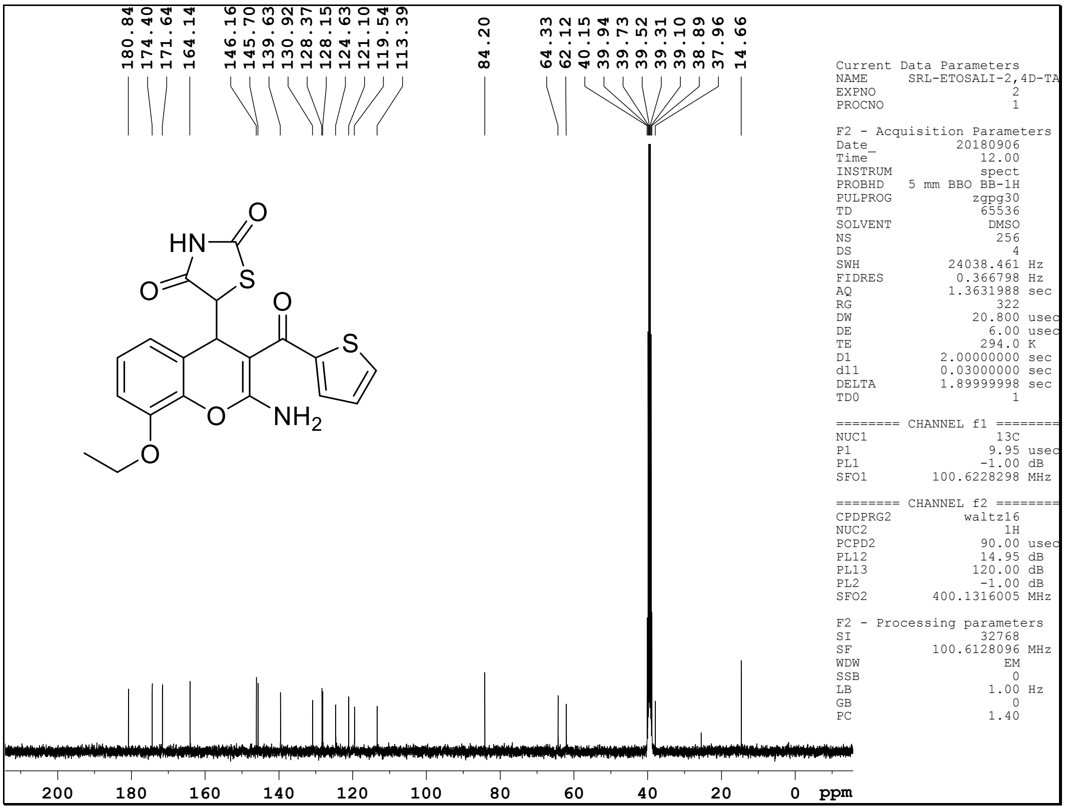

Fig 24: ^{1}H, ^{13}C spectrum of 5-(2-amino-8-ethoxy-3-(thiophene-2-carbonyl)-4H-chromen-4-yl)thiazolidine-2,4-dione **7j**

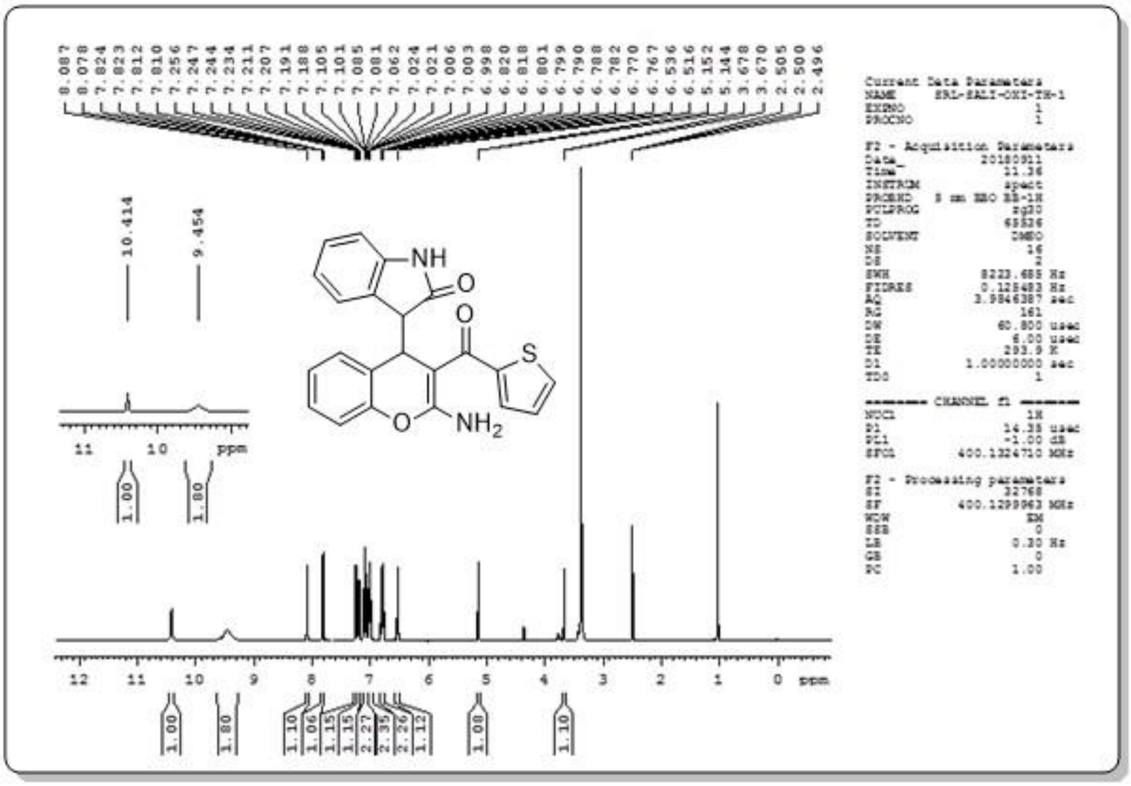

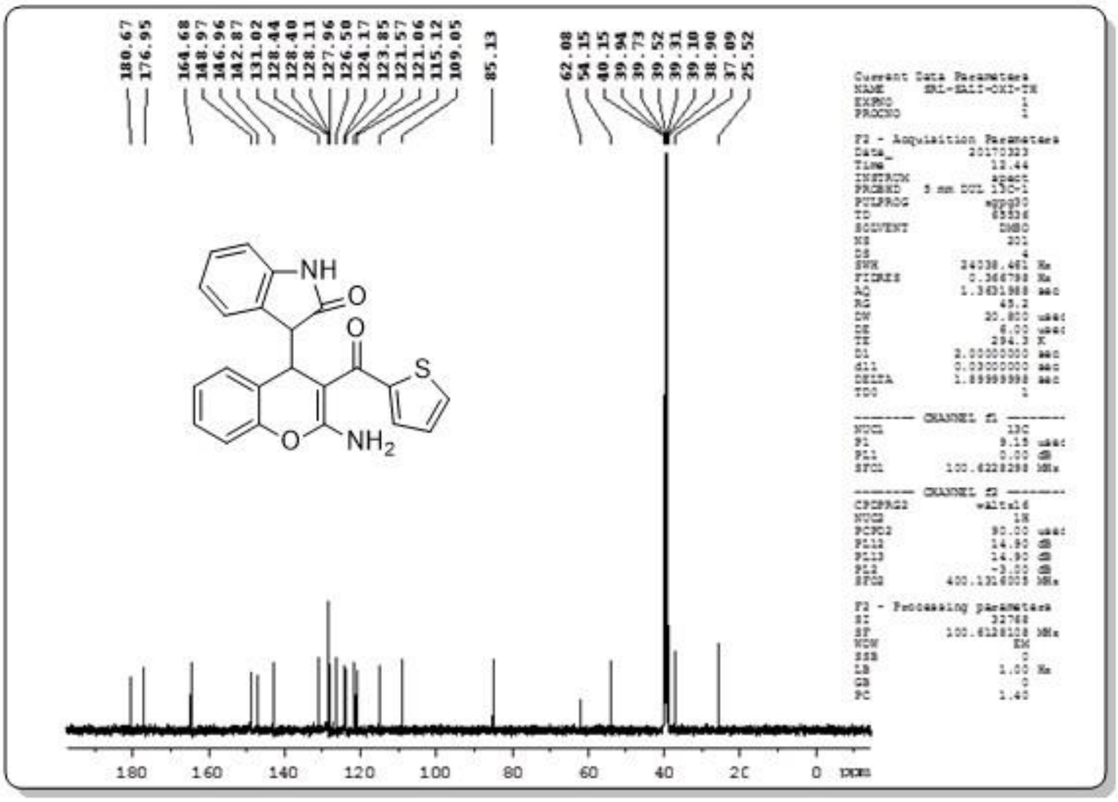

Fig 25: ^{1}H, ^{13}C spectrum of 3-(2-amino-3-(thiophene-2-carbonyl)-4H-chromen-4-yl)indolin-2-one **7k**

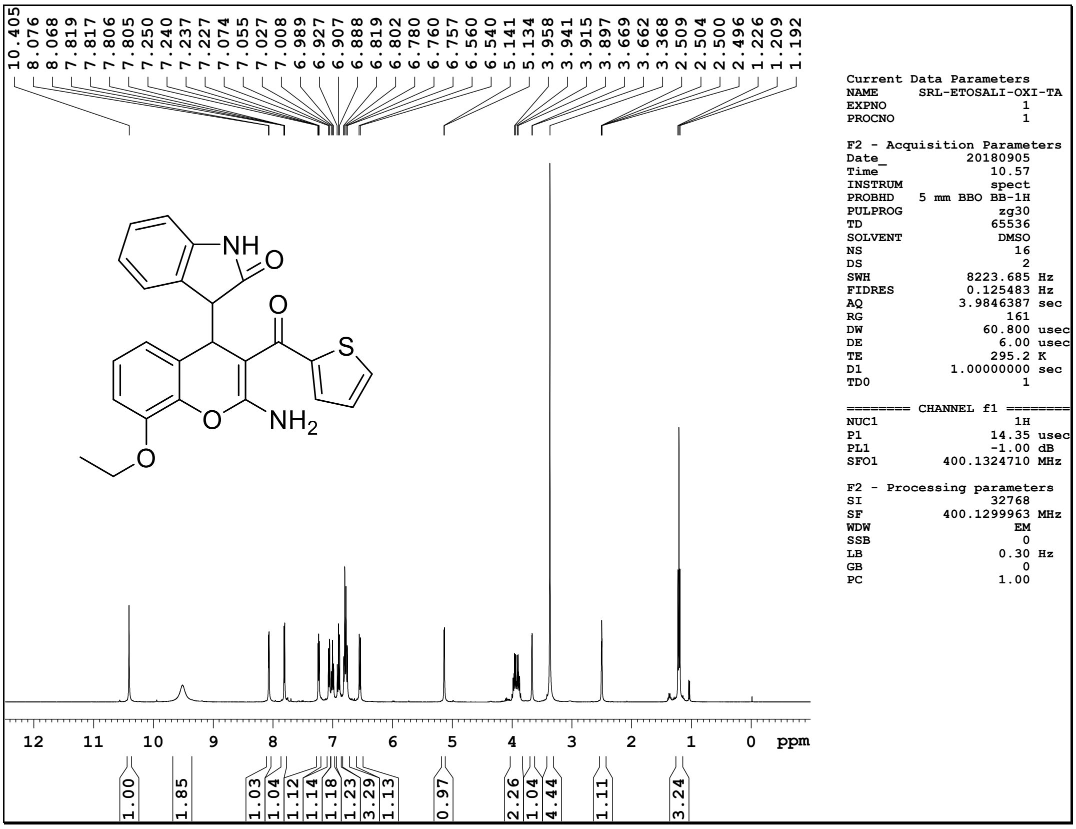

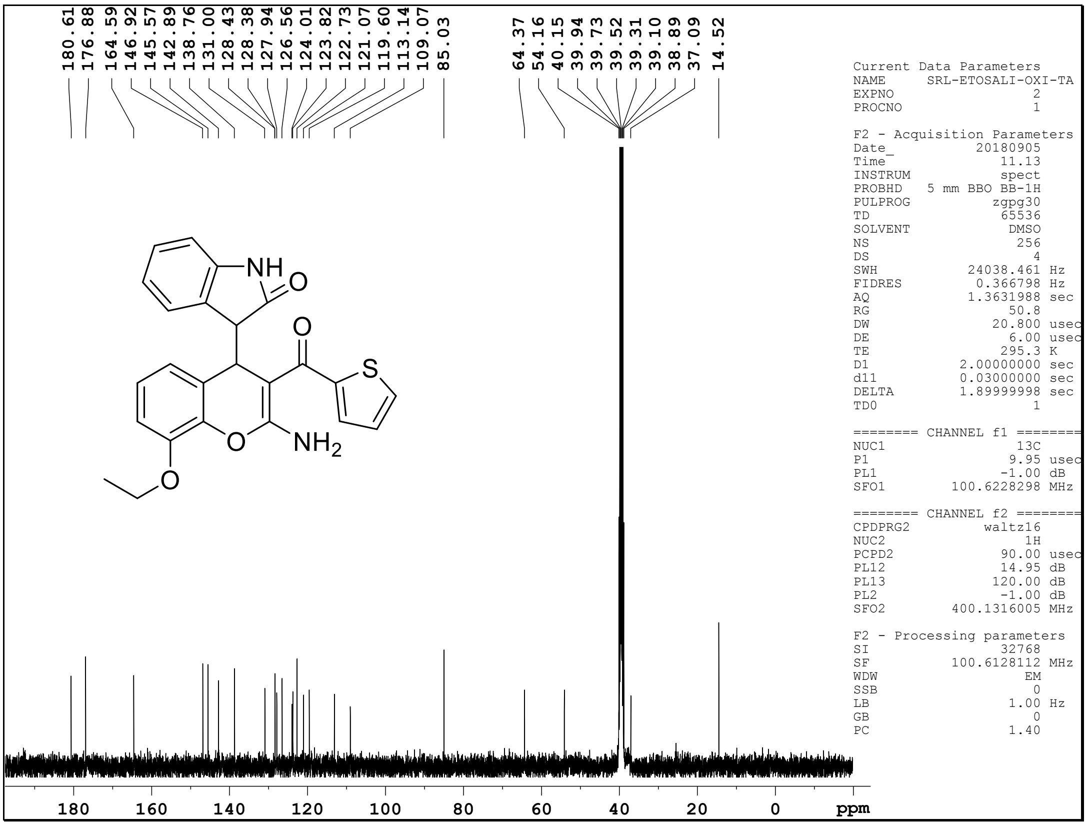

Fig 26: ^{1}H, ^{13}C spectrum of 3-(2-amino-8-ethoxy-3-(thiophene-2-carbonyl)-4H-chromen-4-yl)indolin-2-one **7l**

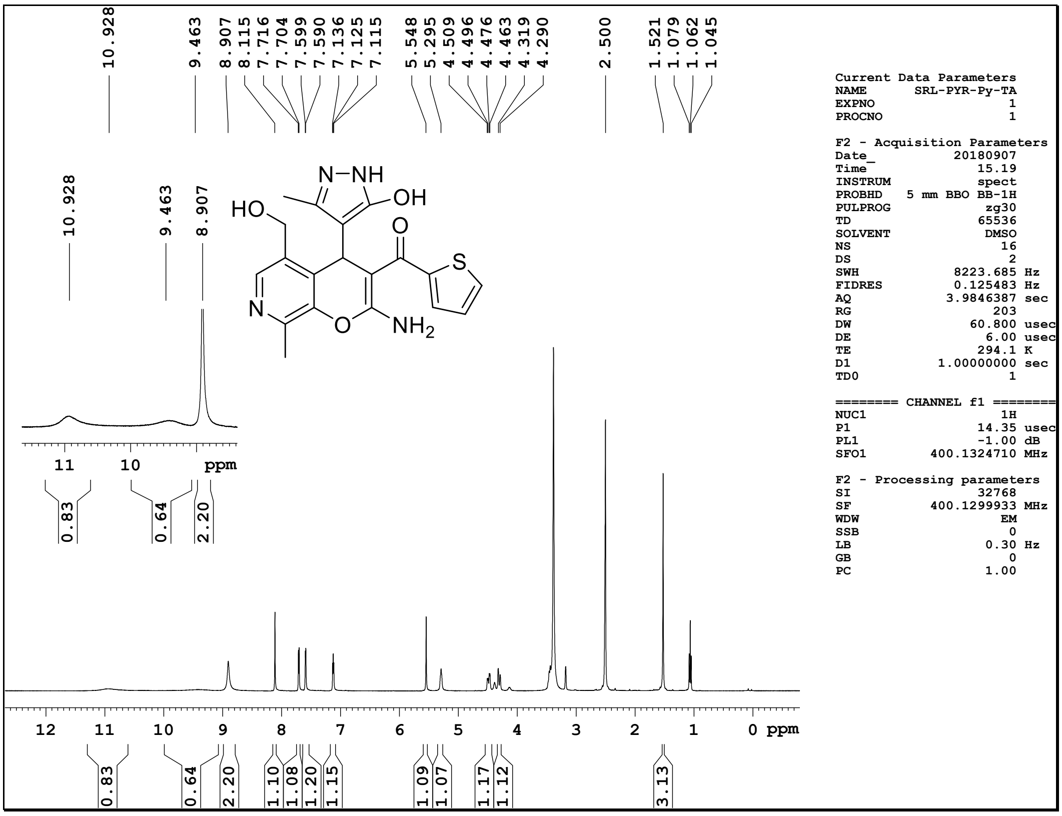

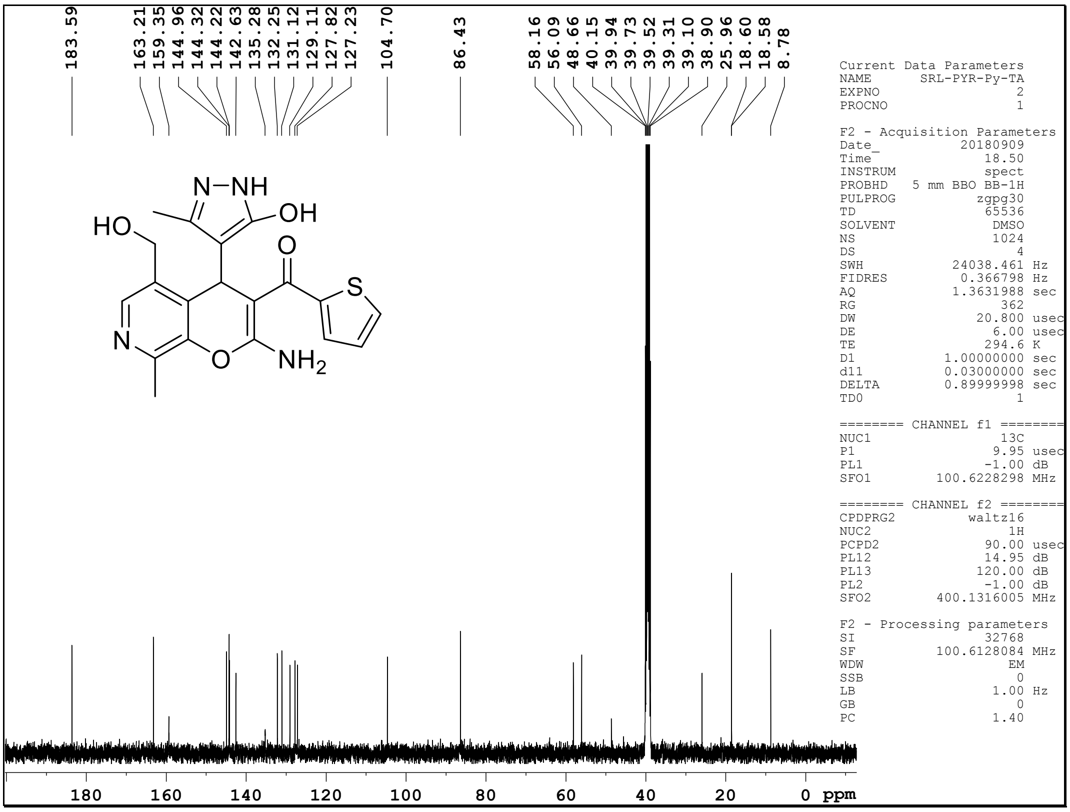

Fig 27: ^{1}H, ^{13}C spectrum of (2-amino-4-(5-hydroxy-3-methyl-1H-pyrazol-4-yl)-5-(hydroxymethyl)-8-methyl-4H-pyrano[2,3-c]pyridin-3-yl)(thiophen-2-yl)methanone **7m**

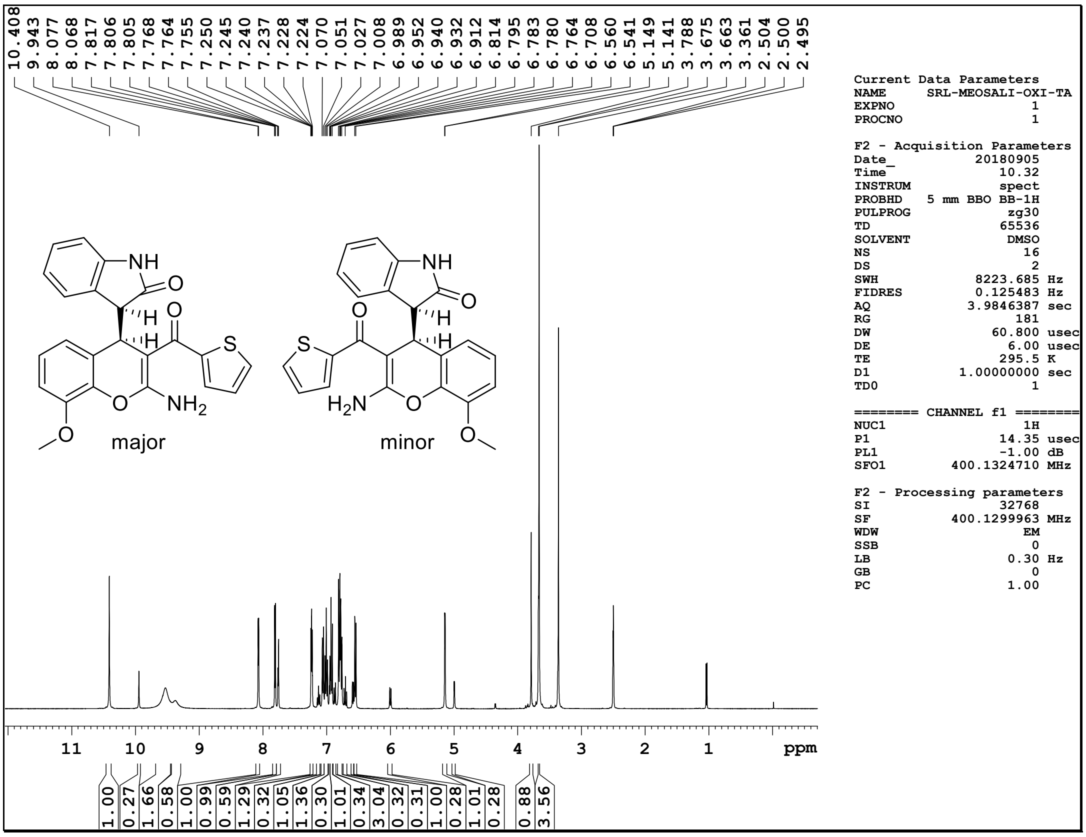

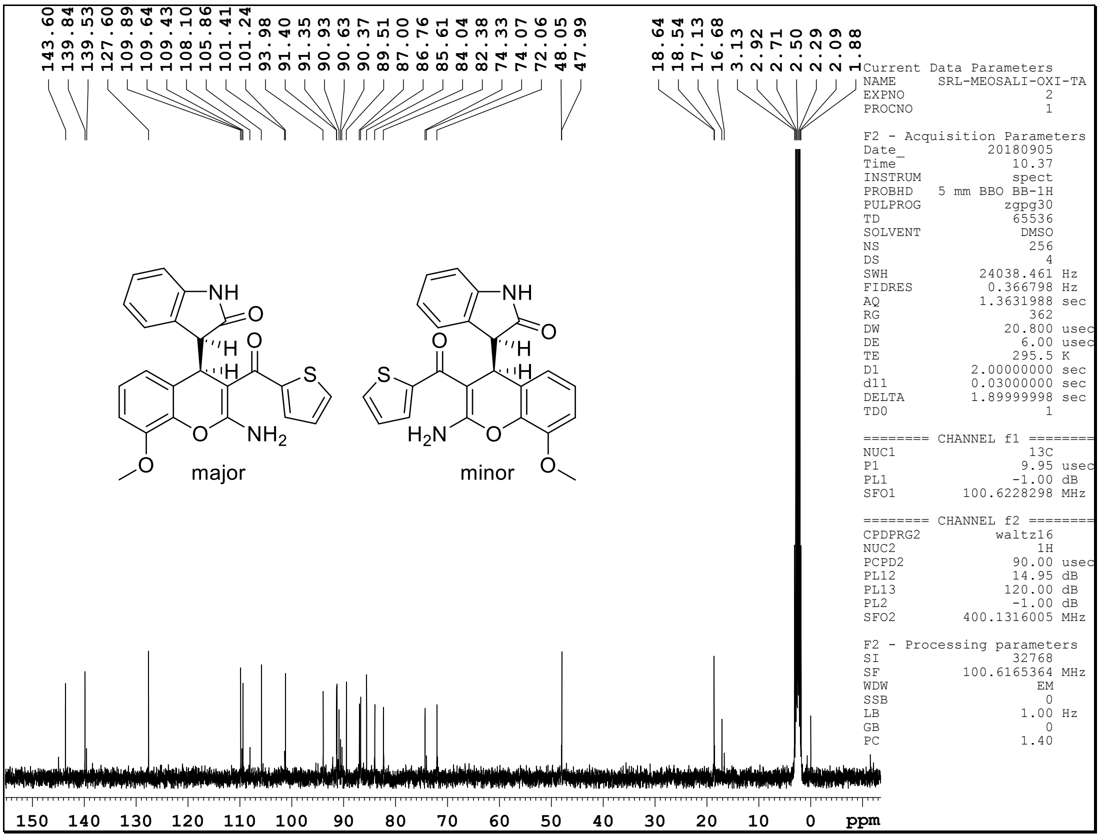

Fig 27: ^{1}H, ^{13}C spectrum of 3-(2-amino-8-methoxy-3-(thiphene-2-carbonyl)-4*H*-chromen-4-yl)indolin-2-one **7n**
